# Supplementary material for: From Bench to Bioactivity: An Integrated Medicinal Development Based on Kinetic and Simulation Assessment of Pyrazolone-Oxadiazole Coupled Benzamide as Promising Inhibitors of Diabetes Mellitus
Source: Pharmaceuticals (Basel). 2025 Oct 22;18(11):1595. doi: 10.3390/ph18111595 (PMC12655744; doi:10.3390/ph18111595)
Supplement: Supplementary file 1 [file pharmaceuticals-18-01595-s001.zip › pharmaceuticals-3908728-supplementary-updated.pdf]

pharmaceuticals

MDPI

Supplementary Materials: From Bench to Bioactivity: An Integrated Medicinal Development Based on Kinetic and Simulation Assessment of Pyrazolone-Oxadiazole Coupled Benzamide as Promising Inhibitors of Diabetes Mellitus

M.M. Khowdiary and Shifa Felemban

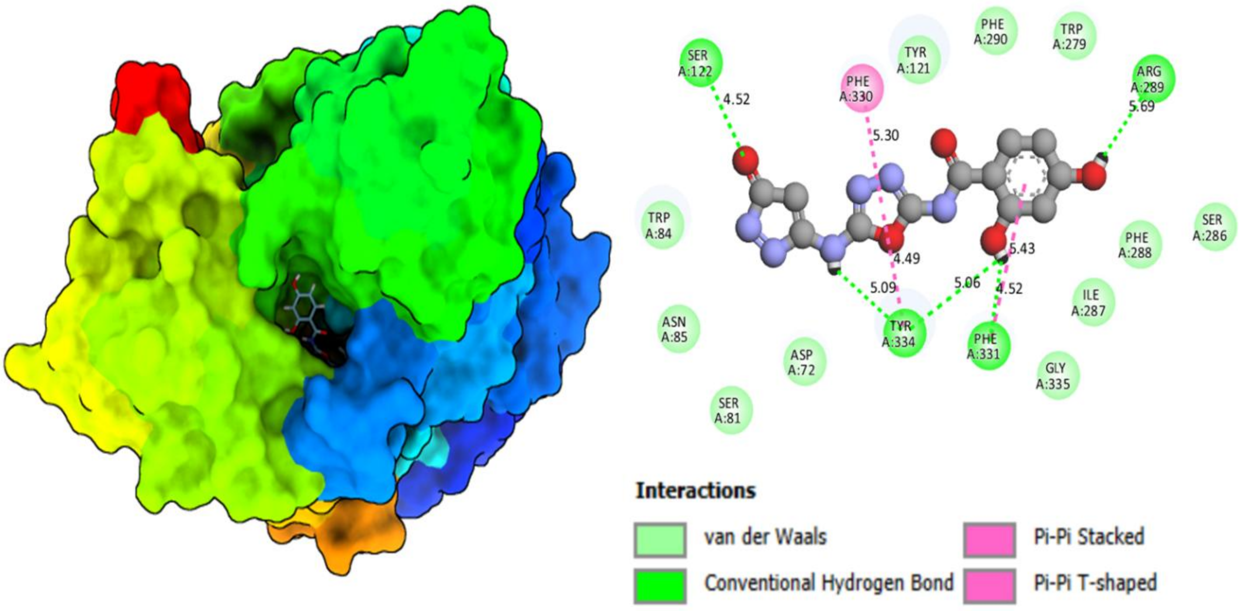

Figure S1. Binding interactions pattern of compound **10** in alpha-amylase complex.

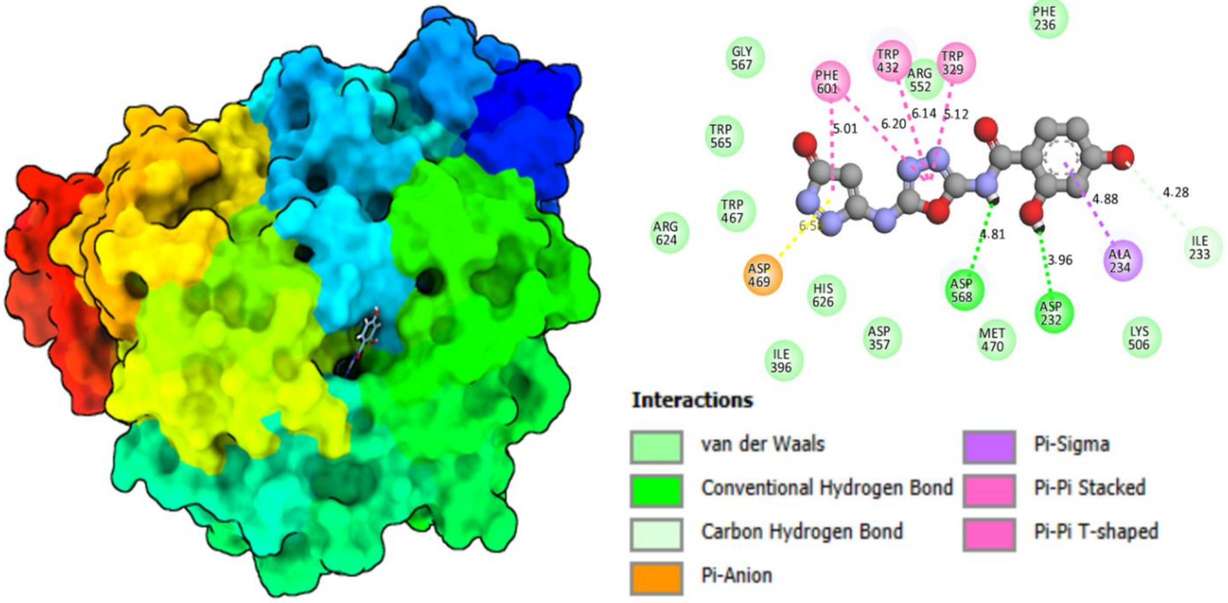

Figure S2. Binding interactions pattern of compound **10** in alpha-glucosidase complex.

Table S1. Various binding mode of interactions of analog **3** and **10**.

Active Analogues	Receptor	Type of Interaction	Distance (A^0)	Docking Score
Compound 3 in alpha-amylase	ILE-A-287	Carbon-Hydrogen bond	4.52	−7.62
	TYR-A-334	Pi-Pi T shaped	5.50	
	PHE-A-330	Pi-Pi T shaped	5.74	
	GLU-A-199	C-H bond	5.18	
	SER-A-200	C-H bond	4.65	
	TYR-A-121	C-H bond	6.65	
	PHE-A-331	Carbon-Hydrogen bond	4.13	
Compound 3 in alpha-glucosidase	ASP-568	Pi-Anion	3.30	−8.14
	TRP-565	C-H bond	4.91	
	HIS-626	Pi-Pi T-shaped	6.04	
	MET-470	Pi-Alkyl	6.19	
	PHE-601	Pi-Pi T-shaped	6.40	
	TRP-432	Pi-Pi T-shaped	6.94	
	TRP-329	Pi-Pi T-shaped	5.85	
	ASP-232	C-H bond	4.08	
	ARG-552	Unfavorable Donar-Donar	6.20	
	ARG-552	Unfavorable Donar-Donar	6.30	
Compound 10 in alpha-amylase	SER-A-122	C-H bond	4.52	−7.68
	PHE-A-330	Pi-Pi stacked	5.30	
	ARG-A-289	C-H bond	5.69	
	PHE-A-331	C-H bond	4.52	
	PHE-A-331<br>TYR-A-334	Pi-Pi stacked<br>C-H bond	5.43<br>5.09	
	TYR-A-334	C-H bond	5.06	
	PHE-601	Pi-Pi stacked	5.01	-8.36

Compound 10 in alpha-glucosidase	PHE-601	Pi-Pi stacked	6.20
	TRP-432	Pi-Pi stacked	6.14
	TRP-329	Pi-Pi T-stacked	5.12
	ILE-233	Carbon –Hydrogen bond	4.28
	ALA-234	Pi-Sigma	4.88
	ASP-232	C-H bond	3.96
	ASP-568	C-H bond	4.81
	ASP-469	Pi-Anion	6.58

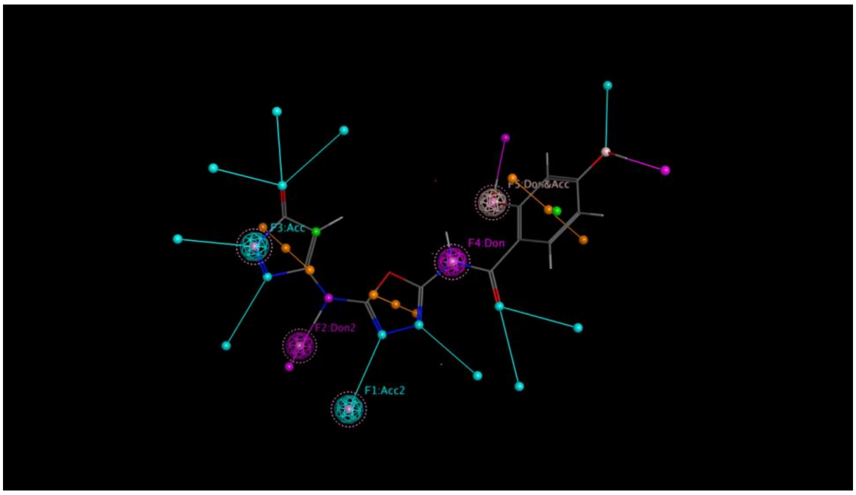

Figure S3. Pharmacophore model output of analog **10** in alpha-amylase.

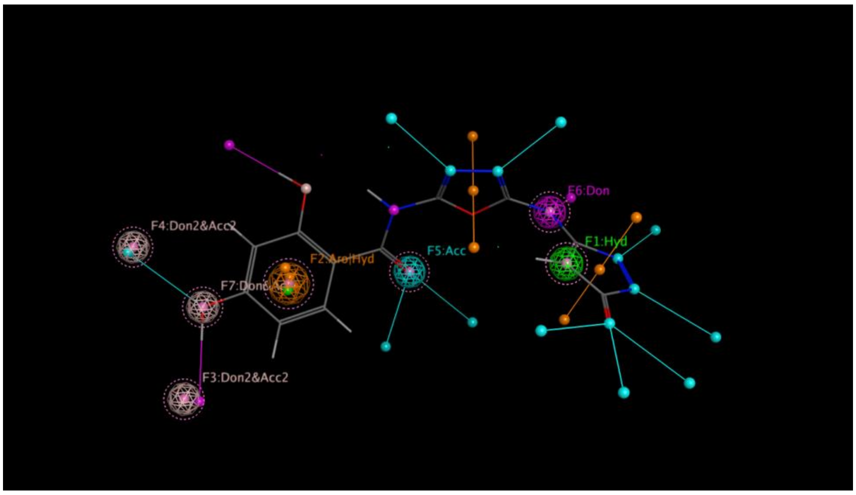

Figure S4. Pharmacophore model output of analog **10** in alpha-glucosidase.

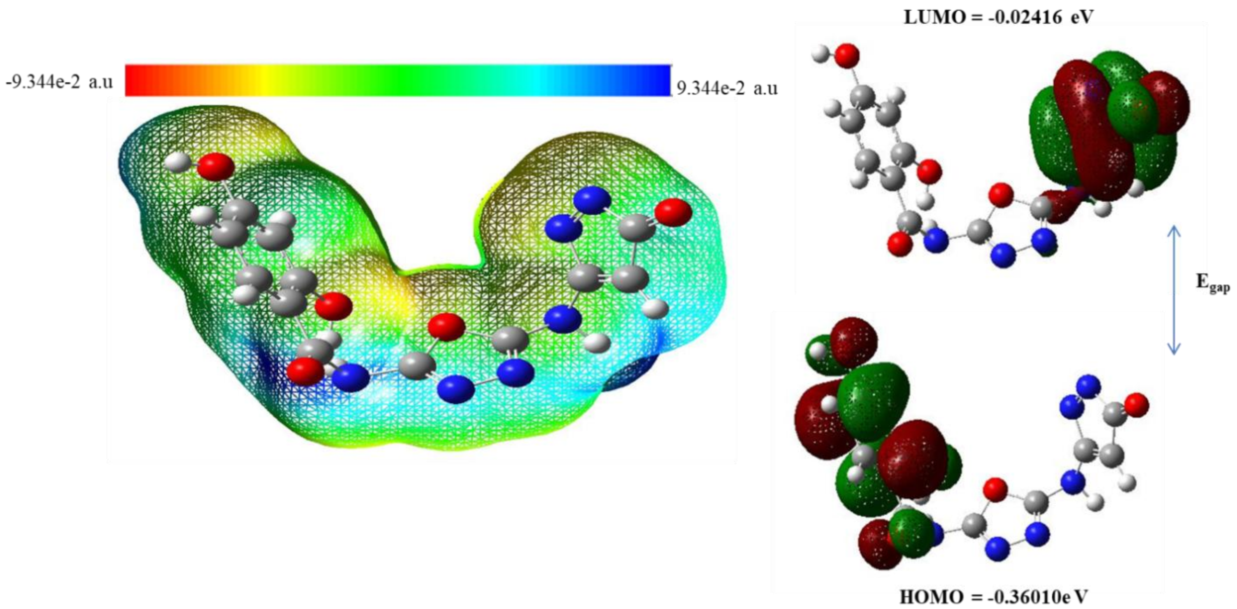

Figure S5. Electronic properties (MESP) on left side and HOMO-LUMO energy gap on right side of compound **10**.

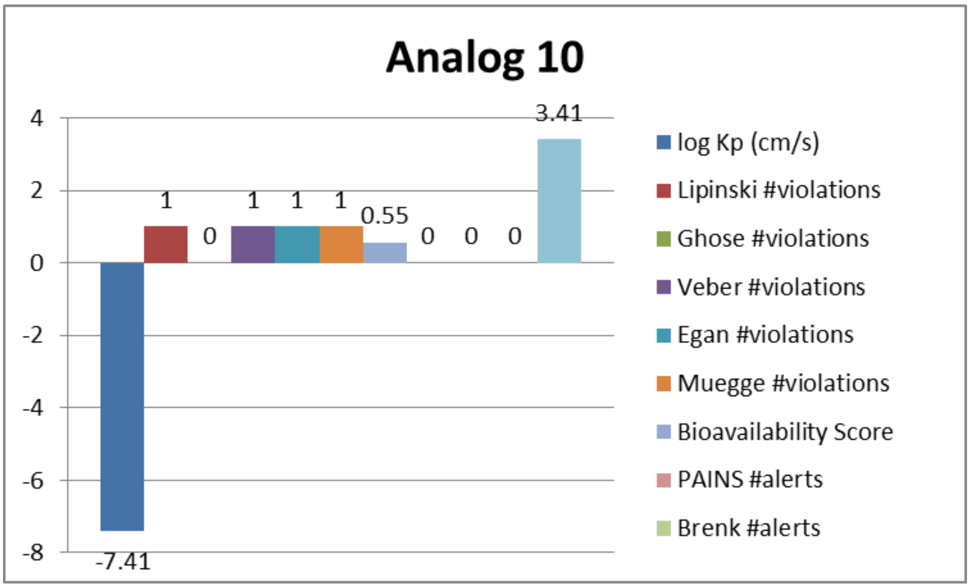

Figure S6. ADMET investigations of analog **10**.

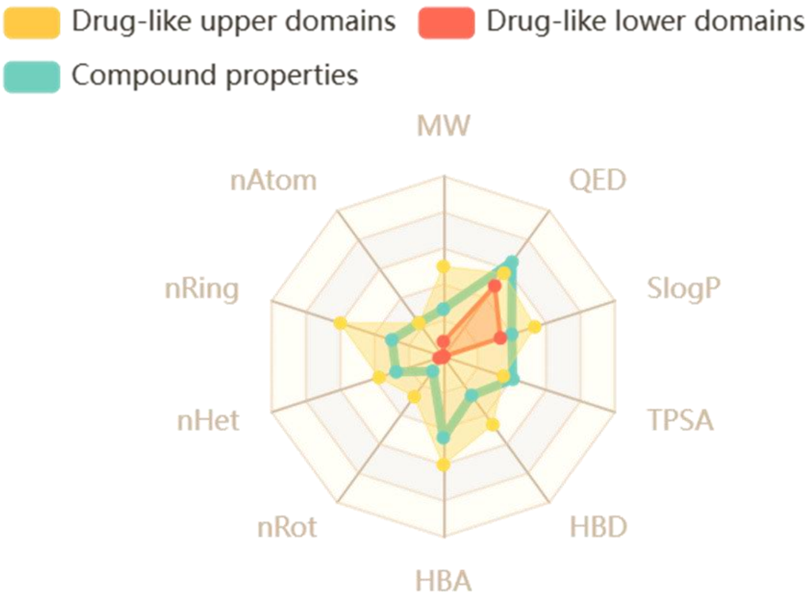

Figure S7. Web sketch of analog **10** showing its characteristics properties.

Table S2. ADMET properties of compound 10.

Property	Model Name	Predicted Value	Unit
Absorption	Water solubility	-2.723	Numeric (log mol/L)
	Caco2 permeability	0.141	Numeric (log Papp in 10^{-6} cm/s)
	Intestinal absorption (human)	71.241	Numeric (% Absorbed)
	Skin Permeability	-2.777	Numeric (log Kp)
	P-glycoprotein substrate	Yes	Categorical (Yes/No)
	P-glycoprotein I inhibitor	No	
	P-glycoprotein II inhibitor		
Distribution	VDss (human)	-0.153	Numeric (log L/kg)
	Fraction unbound (human)	0.361	Numeric (Fu)
	BBB permeability	-1.561	Numeric (log BB)
	CNS permeability	-3.674	Numeric (log PS)
Metabolism	CYP2D6 substrate	No	Categorical (Yes/No)
	CYP3A4 substrate		
	CYP1A2 inhibition		
	CYP2C19 inhibition		
	CYP2C9 inhibition		
	CYP2D6 inhibition		
	CYP3A4 inhibition		
Excretion	Total Clearance	-0.057	Numeric (log mL/min/kg)
	Renal OCT2 substrate	No	Categorical (Yes/No)
Toxicity	AMES toxicity		
	Max. tolerated dose (human)	-0.11	Numeric (log mg/kg/day)
	hERG I inhibitor	No	Categorical (Yes/No)
	hERG II inhibitor		
	Oral Rat Acute Toxicity (LD50)	1.827	Numeric (mol/kg)
Toxicity	Oral Rat Chronic Toxicity (LOAEL)	2.328	Numeric (log mg/kg_bw/day)
	Hepatotoxicity	No	Categorical (Yes/No)
	Skin Sensitisation	No	
	T.Pyriformis toxicity	0.311	Numeric (log ug/L)
	Minnow toxicity	3.288	Numeric (log mM)

Table S3. ADMET properties of compound 3.

Property	Model Name	Predicted Value	Unit
Absorption	Water solubility	-2.613	Numeric (log mol/L)
	Caco2 permeability	0.185	Numeric (log Papp in 10^{-6} cm/s)
	Intestinal absorption (human)	64.876	Numeric (% Absorbed)
	Skin Permeability	-2.736	Numeric (log Kp)
	P-glycoprotein substrate	Yes	Categorical (Yes/No)
	P-glycoprotein I inhibitor	No	
	P-glycoprotein II inhibitor		
Distribution	VDss (human)	-0.186	Numeric (log L/kg)
	Fraction unbound (human)	0.276	Numeric (Fu)
	BBB permeability	-1.914	Numeric (log BB)
	CNS permeability	-4.475	Numeric (log PS)
Metabolism	CYP2D6 substrate	No	Categorical (Yes/No)
	CYP3A4 substrate		
	CYP1A2 inhibitor		
	CYP2C19 inhibitor		
	CYP2C9 inhibitor		
	CYP2D6 inhibitor		
	CYP3A4 inhibitor		
Excretion	Total Clearance	0.03	Numeric (log mL/min/kg)
	Renal OCT2 substrate	No	Categorical (Yes/No)
Toxicity	AMES toxicity		
	Max. tolerated dose (human)	0.412	Numeric (log mg/kg/day)
	hERG I inhibitor	No	Categorical (Yes/No)
	hERG II inhibitor		
	Oral Rat Acute Toxicity (LD50)	2.152	Numeric (mol/kg)
	Oral Rat Chronic Toxicity (LOAEL)	2.852	Numeric (log mg/kg_bw/day)
	Hepatotoxicity	No	Categorical (Yes/No)
	Skin Sensitisation	No	
	T.Pyriformis toxicity	0.286	Numeric (log ug/L)
	Minnow toxicity	2.835	Numeric (log mM)

S1. Spectral analysis

Potassium (3-oxo-3H-pyrazol-5-yl)carbamodithioate (Intermediate II)

^{1}H NMR (600 MHz, DMSO-d_6): δ 8.67 (s, 1H, H-NH), 6.30 (s, 1H, H-Pyrazolone); ^{13}C NMR (150 MHz, DMSO-d_6): δ 194.8, 163.6, 155.6, 120.2, HREI MS: *m*/*z* calcd for $C_4H_2KN_3OS_2$ $[M]^+$ 210.93 Found 210.05.

5-((5-amino-1,3,4-oxadiazol-2-yl)amino)-3H-pyrazol-3-one (Intermediate III)

^{1}H NMR (600 MHz, DMSO-d_6): δ 9.43 (s, 1H, H-NH), 7.67 (s, 2H, H-NH_2), 5.97 (s, 1H, H-pyrazolone); ^{13}C NMR (150 MHz, DMSO-d_6): δ 170.6, 169.7, 168.5, 160.1, 115.2, HREI MS: *m*/*z* calcd for $C_5H_4N_6O_2$ $[M]^+$ 180.04 Found 179.05.

4-fluoro-3-methyl-N-(5-((3-oxo-3H-pyrazol-5-yl)amino)-1,3,4-oxadiazol-2-yl) benzamide (1)

Physical state: amorphous solid (powder), color: light green, M.P: 231-232 °C, ^{1}H NMR (600MHz, DMSO-*d$_6$*): *δ* 12.30 (s, 1H, H-amide), 9.41 (s, 1H, H-NH), 8.08 (d, *J* = 7.4 Hz, 1H, H-Ar), 7.74 (s, 1H, H-Ar), 7.34 (d, *J* = 7.3 Hz, 1H, H-Ar), 5.89 (s, 1H, H-pyrazolone), 2.35 (s, 3H, H-aliphatic); ^{13}C NMR (150 MHz, DMSO-*d$_6$*): *δ* 169.1, 168.1, 167.4, 164.0, 162.6, 160.9, 129.3, 126.1, 124.0, 120.2, 115.5, 112.2, 14.1; HREI MS:*m/z calcd* for $C_{13}H_9FN_6O_3$ [M]$^+$ 316.07 FOUND 316.05.

3,5-dimethyl-N-(5-((3-oxo-3H-pyrazol-5-yl) amino)-1,3,4-oxadiazol-2-yl) benzamide (2)

Physical state: amorphous solid (powder), color: yellow, M.P: 201-202 °C, ^{1}H NMR (600MHz, DMSO-*d$_6$*): *δ* 12.43 (s, 1H, H-amide), 9.41 (s, 1H, H-NH), 7.70 (s, 2H, H-Ar), 7.57 (s, 1H, H-Ar), 5.87 (s, 1H, H-pyrazolone), 2.34 (s, 6H, H-aliphatic); ^{13}C NMR (150 MHz, DMSO-*d$_6$*): *δ* 169.3, 168.2, 167.2, 164.7, 159.3, 139.1, 136.4, 134.3, 133.2, 128.4, 127.9, 115.5, 21.2, 20.3; HREI MS:*m/z calcd* for $C_{14}H_{12}N_6O_3$ [M]$^+$ 312.10 FOUND 312.09.

2,4,6-trihydroxy-N-(5-((3-oxo-3H-pyrazol-5-yl)amino)-1,3,4-oxadiazol-2-yl) benzamide (3)

Physical state: amorphous solid (powder), color: green, M.P: 236-238 °C, ^{1}H NMR (600MHz, DMSO-*d$_6$*): *δ* 14.38 (s, 2H, H-OH), 12.41 (s, 1H, H-amide), 10.53 (s, 1H, H-OH), 9.48 (s, 1H, H-NH), 6.89 (s, 2H, H-Ar), 5.98 (s, 1H, H-pyrazolone); ^{13}C NMR (150 MHz, DMSO-*d$_6$*): *δ* 169.3, 168.2, 167.2, 164.7, 163.1, 161.2, 160.3, 159.3, 115.5, 101.1, 96.3, 94.2; HREI MS:*m/z calcd* for $C_{12}H_8N_6O_6$ [M]$^+$ 332.05 FOUND 332.04.

2-fluoro-N-(5-((3-oxo-3H-pyrazol-5-yl) amino)-1,3,4-oxadiazol-2-yl) benzamide (4)

Physical state: amorphous solid (powder), color: yellow, M.P: 227-228 °C, ^{1}H NMR (600MHz, DMSO-*d$_6$*): *δ* 12.43 (s, 1H, H-amide), 9.48 (s, 1H, H-NH), 8.14 (dd, *J* = 7.4, 2.3 Hz, 1H, H-Ar), 7.51 (dd, *J* = 7.3, 2.1 Hz, 1H, H-Ar), 7.29 (M, 1H, H-Ar), 7.15 (M, 1H, H-Ar), 5.97 (s, 1H, H-pyrazolone); ^{13}C NMR (150 MHz, DMSO-*d$_6$*): δ 169.3, 167.2, 164.7, 160.3, 159.3, 133.7, 131.3, 126.0, 125.1, 124.3, 115.6, 113.5; HREI MS:*m/z calcd* for $C_{12}H_7N_6O_6$ [M]$^+$ 302.06 FOUND 302.05.

3-hydroxy-N-(5-((3-oxo-3H-pyrazol-5-yl) amino)-1,3,4-oxadiazol-2-yl) benzamide (5)

Physical state: amorphous solid (powder), color: light brown, M.P: 245-245 °C, ^{1}H NMR (600MHz, DMSO-*d$_6$*): *δ* 12.43 (s, 1H, H-amide), 9.49 (s, 1H, H-NH), 9.45 (s, 1H, H-OH), 7.56 (dd, *J* = 7.4, 2.3 Hz, 1H, H-Ar), 7.53 (dd, *J* = 7.1, 2.2 Hz, 1H, H-Ar), 7.39 (s, 1H, H-Ar), 7.21 (dd, *J* = 7.2, 2.3 Hz, 1H, H-Ar), 5.99 (s, 1H, H-pyrazolone); ^{13}C NMR (150 MHz, DMSO-*d$_6$*): *δ* 169.3, 168.2, 167.2, 164.7, 160.3, 158.6, 136.2, 130.2, 122.1, 120.1, 119.3, 115.4 HREI MS:*m/z calcd* for $C_{12}H_8N_6O_4$ [M]$^+$ 300.06 FOUND 300.04.

4-cyano-N-(5-((3-oxo-3H-pyrazol-5-yl) amino)-1,3,4-oxadiazol-2-yl) benzamide (6)

Physical state: amorphous solid (powder), color: light green, M.P: 219-221 °C, ^{1}H NMR (600MHz, DMSO-*d$_6$*): *δ* 12.40 (s, 1H, H-amide), 9.44 (s, 1H, H-NH), 8.17 (dd, *J* = 7.2, 2.1 Hz, 2H, H-Ar), 8.12 (dd, *J* = 7.4, 2.3 Hz, 2H, H-Ar), 5.55 (s, 1H, H-pyrazolone); ^{13}C NMR (150

MHz, DMSO-*d_6*): *δ* 169.3, 168.4, 167.2, 164.3, 160.3, 135.2, 133.5, 132.6, 128.2, 127.2, 118.2, 116.0, 115.5 HREI MS:*m/z calcd* for $C_{13}H_7N_7O_3$ $[M]^+$ 309.06 FOUND 309.05.

4-fluoro-3-hydroxy-N-(5-((3-oxo-3H-pyrazol-5-yl)amino)-1,3,4-oxadiazol-2-yl) benzamide (7)

Physical state: amorphous solid (powder), color: yellow, M.P: 244-246 °C, ^1H NMR (600MHz, DMSO-*d_6*): *δ* 12.45 (s, 1H, H-amide), 9.43 (s, 1H, H-NH), 9.01 (s, 1H, H-OH), 7.67 (d, *J* = 7.3 Hz, 1H, H-Ar), 7.31 (s, 1H, H-Ar), 7.22 (D, *J* = 7.2 Hz, 1H, H-Ar), 5.97 (s, 1H, H-pyrazolone); ^{13}C NMR (150 MHz, DMSO-*d_6*): *δ* 169.3, 168.2, 167.2, 164.4, 160.3, 157.1, 146.9, 131.2, 121.8, 117.0, 115.5, 114.8 HREI MS:*m/z calcd* for $C_{12}H_7FN_6O_4$ $[M]^+$ 318.05 FOUND 318.04.

2-chloro-5-methyl-N-(5-((3-oxo-3H-pyrazol-5-yl)amino)-1,3,4-oxadiazol-2-yl) benzamide (8)

Physical state: amorphous solid (powder), color: light green, M.P: 235-237 °C, ^1H NMR (600MHz, DMSO-*d_6*): *δ* 12.49 (s, 1H, H-amide), 9.42 (s, 1H, H-NH), 7.70 (s, 1H, H-Ar), 7.55 (d, *J* = 7.2 Hz, 1H, H-Ar), 7.31 (d, *J* = 7.4 Hz, 1H, H-Ar), 5.96 (s, 1H, H-pyrazolone), 2.42 (s, 3H, H-aliphatic); ^{13}C NMR (150 MHz, DMSO-*d_6*): δ 169.3, 168.2, 167.2, 164.7, 160.3, 136.6, 133.8, 132.2, 131.6, 129.1, 127.7, 115.5, 20.9; HREI MS:*m/z calcd* for $C_{13}H_9ClN_6O_3$ $[M]^+$ 332.04 FOUND 332.03.

4-bromo-N-(5-((3-oxo-3H-pyrazol-5-yl) amino)-1,3,4-oxadiazol-2-yl) benzamide (9)

Physical state: amorphous solid (powder), color: brown, M.P: 248-250 °C, ^1H NMR (600MHz, DMSO-*d_6*): *δ* 12.54 (s, 1H, H-amide), 9.37 (s, 1H, H-NH), 7.95 (d, *J* = 7.4 Hz, 2H, H-Ar), 7.77 (d, *J* = 7.1 Hz, 2H, H-Ar), 5.94 (s, 1H, H-pyrazolone), ^{13}C NMR (150 MHz, DMSO-*d_6*): *δ* 169.6, 168.4, 166.4, 163.0, 160.9, 134.9, 131.7, 130.5, 129.3, 126.3, 122.2, 115.7; HREI MS:*m/z calcd* for $C_{12}H_7BrN_6O_3$ $[M]^+$ 361.98 FOUND 361.97.

2,4-dihydroxy-N-(5-((3-oxo-3H-pyrazol-5-yl) amino)-1,3,4-oxadiazol-2-yl) benzamide (10)

Physical state: amorphous solid (powder), color: light yellow, M.P: 213-214 °C, ^1H NMR (600MHz, DMSO-*d_6*): *δ* 14.43 (s, 1H, H-OH), 12.46 (s, 1H, H-amide), 10.13 (s, 1H, H-OH), 9.49 (s, 1H, H-NH), 7.52 (d, *J* = 7.3 Hz, 1H, H-Ar), 6.43 (d, *J* = 7.4 Hz, 1H, H-Ar), 6.36 (s, 1H, H-Ar), 5.95 (s, 1H, H-pyrazolone); ^{13}C NMR (150 MHz, DMSO-*d_6*): *δ* 169.3, 168.2, 167.2, 164.7, 163.3, 160.8, 159.3, 130.3, 115.5, 112.4, 111.6, 103.7; HREI MS:*m/z calcd* for $C_{12}H_8N_6O_5$ $[M]^+$ 316.06 FOUND 316.04.

S.2 Alpha-Glucosidase Activity Assay:

The inhibitory potential of Alpha-glucosidase was assessed using a well-established method [33,34]. Briefly, a 96-well plate was utilized, and each well received 35 μL of phosphate buffer, 31 μL of the tested compound solution (concentration range: 50–250 μg/mL), and 18 μL of [4-nitrophenyl-*α*-D glucopyranoside(p-NPG)] substrate. This mixture was then incubated at 37 °C for 5 min. Subsequently, 16 μL of *α*-glucosidase (0.15 U/mL) dissolved in sodium phosphate was introduced into each well, resulting in a total volume of

100 μL. The reaction was initiated by adding 100 μL of sodium carbonate (200 mM). The absorbance at 405 nm was measured using a microplate reader. This experimental procedure was conducted in triplicate, and a control group without the tested compound was included for comparison. Acarbose served as the standard reference drug. The percentage inhibition was calculated using the following equation, where "Abs" represents absorbance."

$$\%\ \boldsymbol{inhibition} = \frac{(\boldsymbol{Abs\ control} - \boldsymbol{Abs\ sample})}{\boldsymbol{Abs\ control}} \times \mathbf{100}$$

S.3 Alpha-Amylase Activity Assay:

The inhibitory potential of α-Amylase enzyme was evaluated through the standard methodology [35]. A test tube containing (250 μL) of the compound being tested at various concentrations (50–250 μg/mL), (250 μL) of [1% (*w*/*v*)] starch solution, and (250 μL) of (1U/mL) alpha-amylase solution was prepared. Post-incubation at 20°C for 3 min, the enzymatic process was halted by adding (500 μL) of dinitro salicylic acid (color Reagent). Following this, (250 μL) of α-amylase was immediately introduced after heating the mixture in hot water. The mixture was subsequently subjected to heating at 85 °C for duration of 15 min, followed by a 5-min incubation period at room temperature. (4500 μL) of distilled water was added, resulting in a final volume of (6000 μL). Spectrophotometric analysis at 540 nm was conducted to determine absorbance. A control sample devoid of the test substance was prepared, and Acarbose served as the reference drug. The % inhibition equation is used for calculation.

S.4 Docking protocol:

Protein data bank (PDB) was used a medium for retrieval of crystalline structure, optimizing the structure by the removal water molecules, co-factors and hetero-atoms and computing hydrogen bonds, charges and the missing atoms. Benzothiazole based sulfonamide derivatives used for docking studies were prepared and then optimized by the use of built and Ligand Preparation module implemented in Discovery Studio 2018 (Dassault Systemes BIOVIA, USA). Gold docking tool was used for docking analysis, Ligand preparation involves generating varied tautomer's, bond order assigning and stereochemistry. Furthermore, amylase active site was surrounded by the receptor grid choosing centroid of complexed ligand (Montbretin A). Radius of 12 Å around the Montbretin A binding site was defined for enzyme active site. Accomplishment of docking calculations was achieved using Chem PLP scoring function [36].

S.5 DFT ASSAY:

The geometric parameters and energies were computed by density functional theory at the B3LYP/CEP-631G level of theory, using the GAUSSIAN 98W package of the programs [37], on geometries that were optimized at CEP-631G basis set. The high basis set was chosen to detect the energies at a highly accurate level. The atomic charges were computed using the natural atomic orbital populations. The B3LYP is the key word for the hybrid functional [38], which is a linear combination of the gradient functionals proposed by Becke [39] and Lee, Yang and Parr [40], together with the Hartree-Fock local exchange function [41].

S.6 Enzyme kinetic studies:

The kinetic studies were performed by using varying concentrations (μM) which were pre-incubated with test enzymes (250 μL) (3 U/mL) solution at 37 °C for 10 min. In another set of test tubes, test enzyme was pre-incubated with phosphate buffer (250 μL)

(100 mM, pH 6.8), then varying concentrations (0.0–8.0 mM) starch was added to both reaction mixtures which were incubated at 37 °C for 10 min. Afterward, DNS (100 µL) was added to the mixtures for quenching the reaction and the mixtures boiled for 10 min then absorbance was measured using a spectrometer at 540 nm using a maltose standard curve and converted to reaction velocities [42].

S.7 Molecular Dynamics (MD) Simulation:

MD simulation of the best scoring ligand was performed in complex with their respective targeted enzymes in triplicates [43,44]. The protein–ligand complex obtained in the AutoDock Vina is imported to the Maestro interface of the Schrodinger's software. Prior to MD simulation, the protein–ligand complex was optimized by adding missing hydrogen atoms, assigning proper protonation state of the ligand and other parameters using Protein preparation wizard. The protein–ligand complex was placed at the center of an orthorhombic box, keeping a distance of at least 10 Å from the sides of the box. TIP3P water molecules were added to solvate the simulation box, and proper counterions were also added to neutralize the system. The physiological conditions were mimicked by adding 150 mM NaCl. The energy of the whole system was minimized with 2000 iteration and convergence criteria of 1 kcal/mol/Å, using OPLS3e forcefield. The production MD simulation run was performed for 100 ns employing NPT ensemble at 298 K and 1 bar. Temperature and pressure were maintained with the help of Nose-Hoover Chain thermostat and Matrtyna–Tobias–Klein barostate [45,46]. A 2 fs time step was fixed, and at every 10 ps, energies and structures were documented in the trajectory. The parameters such as root mean square deviation (RMSD), root mean square fluctuation (RMSF), radius of gyration (Rg), solvent accessible surface area (SASA), secondary structure analysis, and protein–ligand interactions were analyzed to establish the stability of protein–ligand complexes. The results are presented as mean ± standard deviation of the three independent experiments.

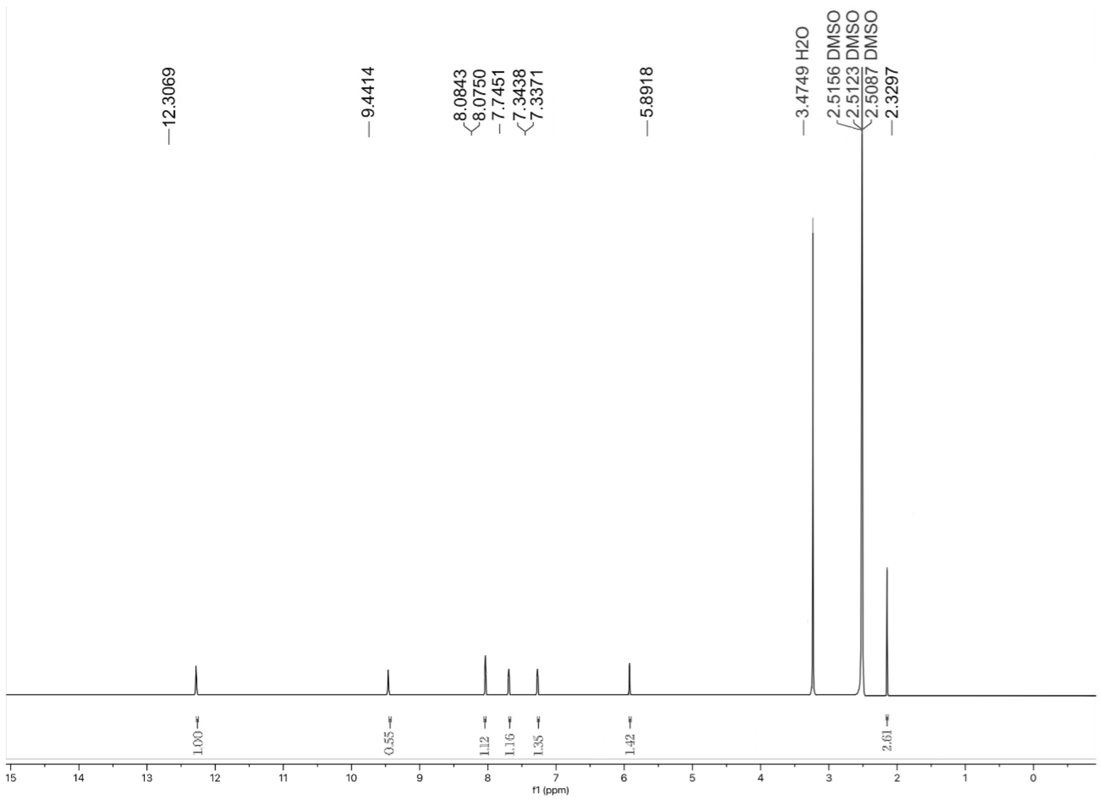

Figure-S.8 Proton spectral analysis of compound-1

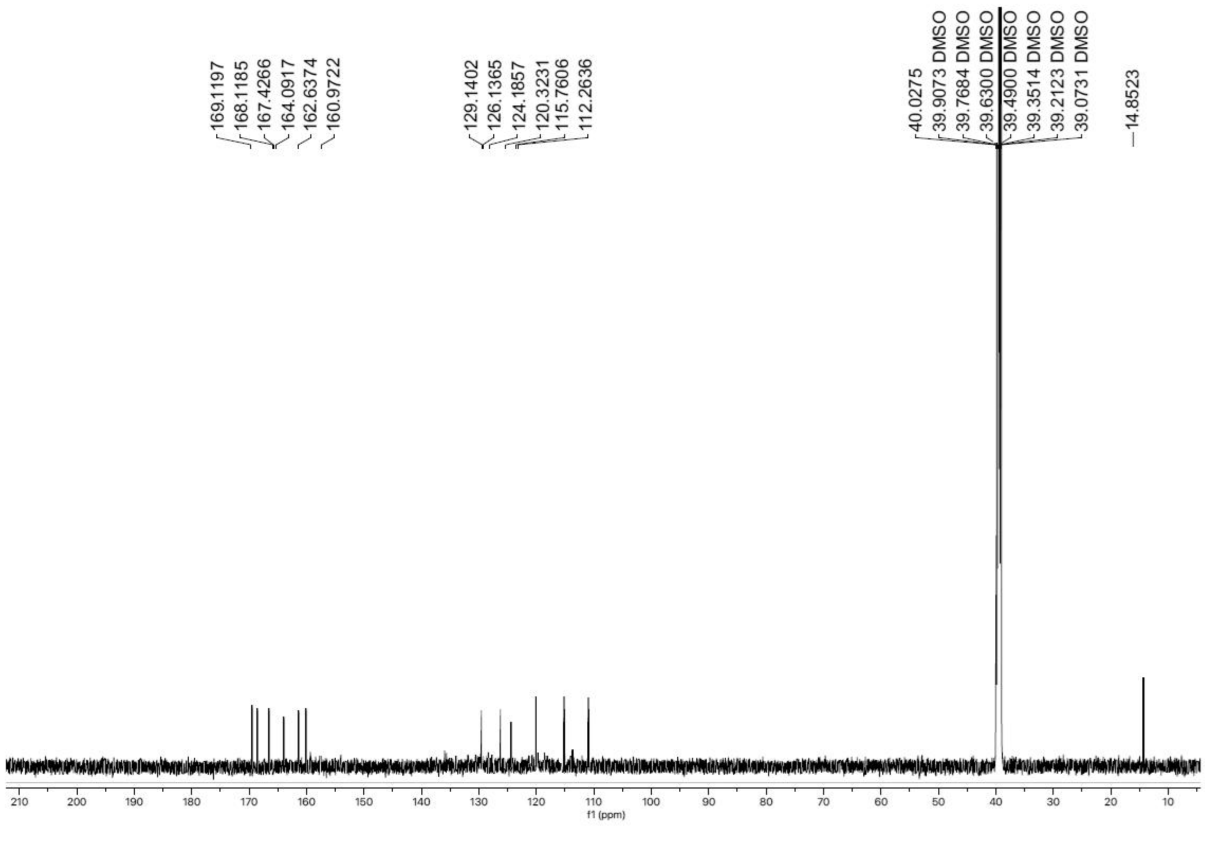

Figure-S.9 Carbon spectral analysis of compound-1

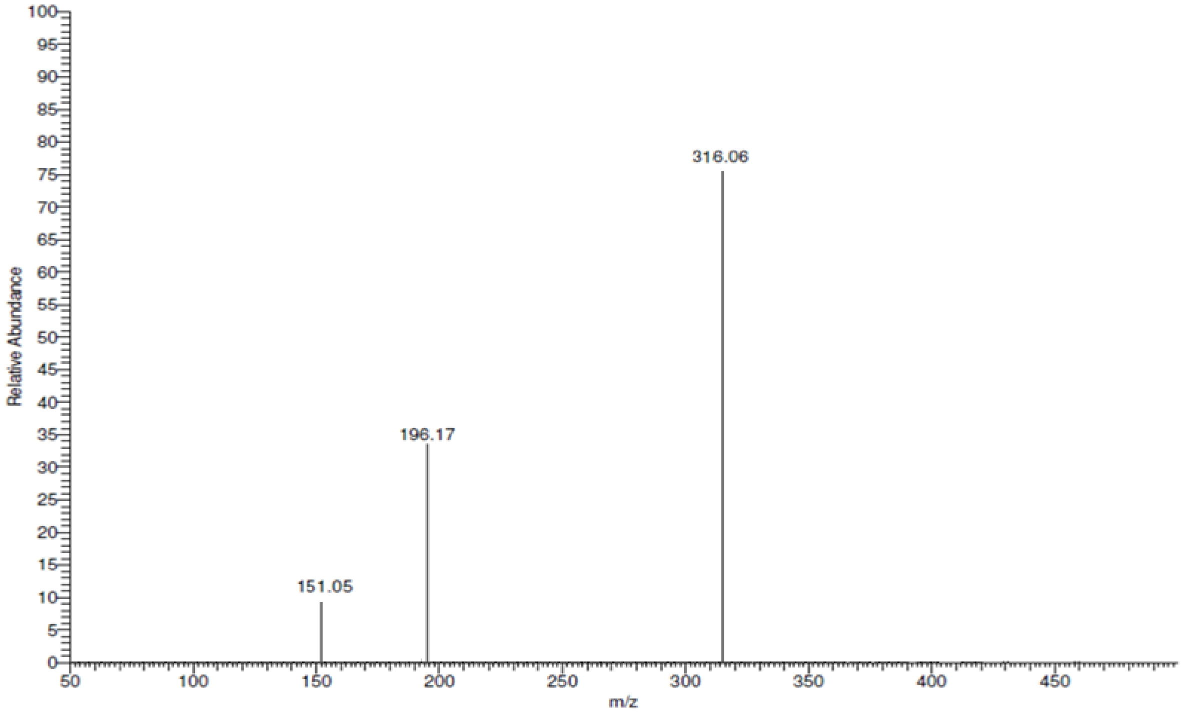

Figure-S.10 HREI-MS spectral analysis of compound-**1**

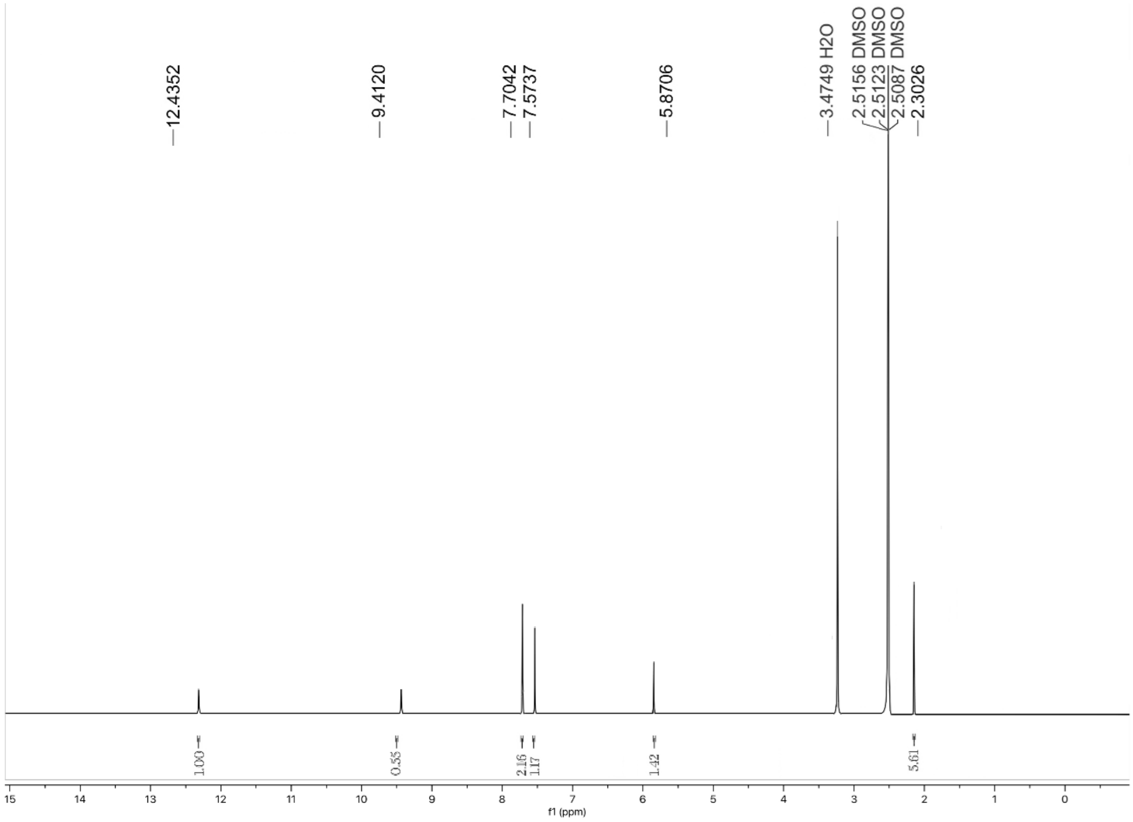

Figure-S.11 Proton spectral analysis of compound-**2**

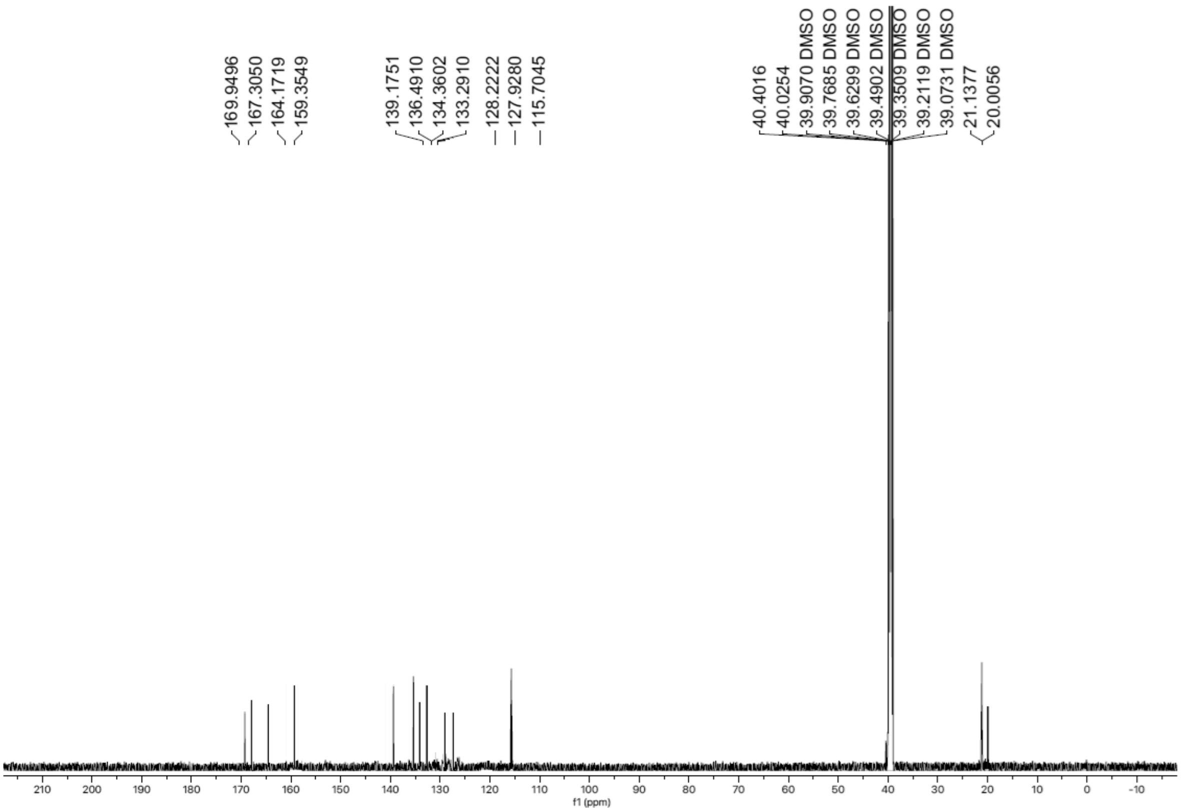

Figure-S.12 Carbon spectral analysis of compound-**2**

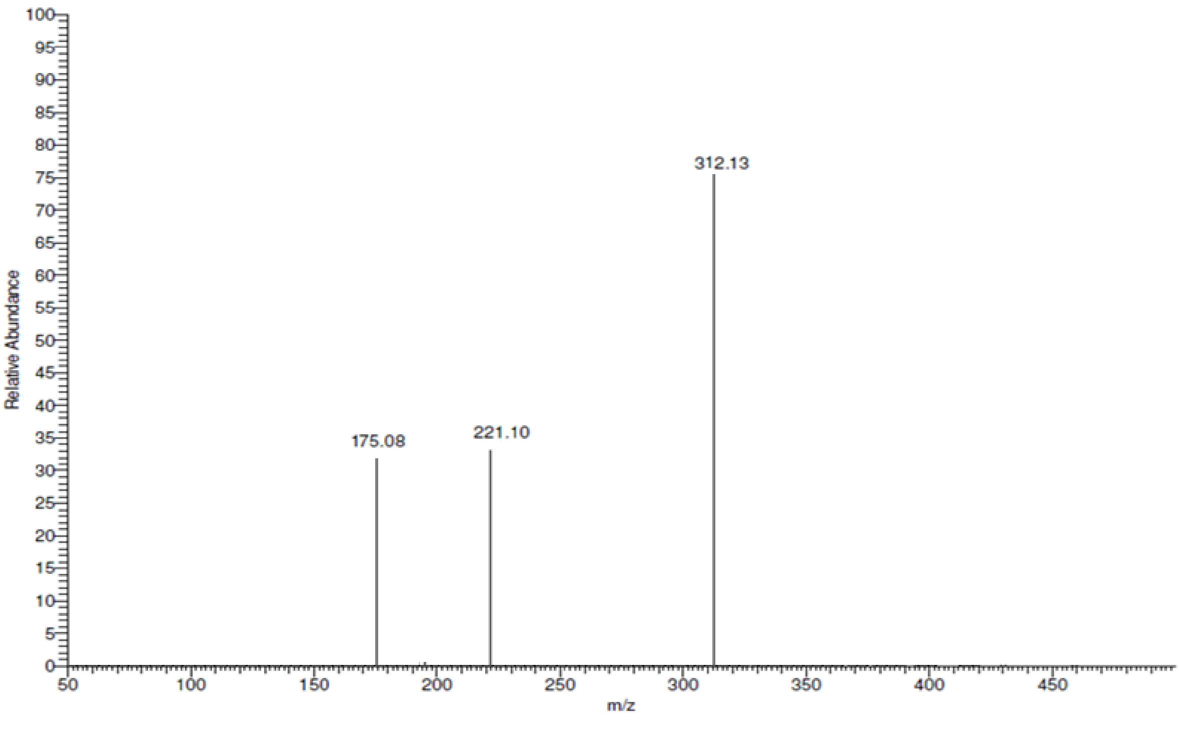

Figure-S.13 HREI-MS spectral analysis of compound-**2**

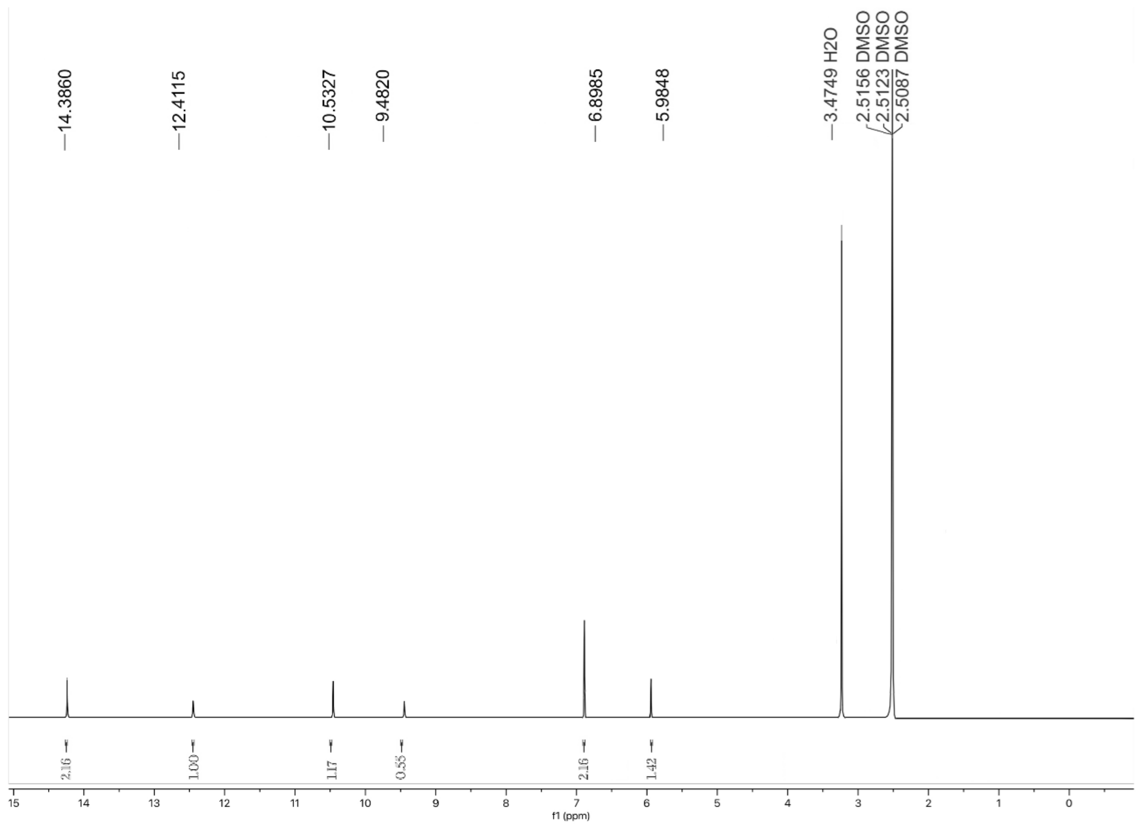

Figure-S.14 Proton spectral analysis of compound-**3**

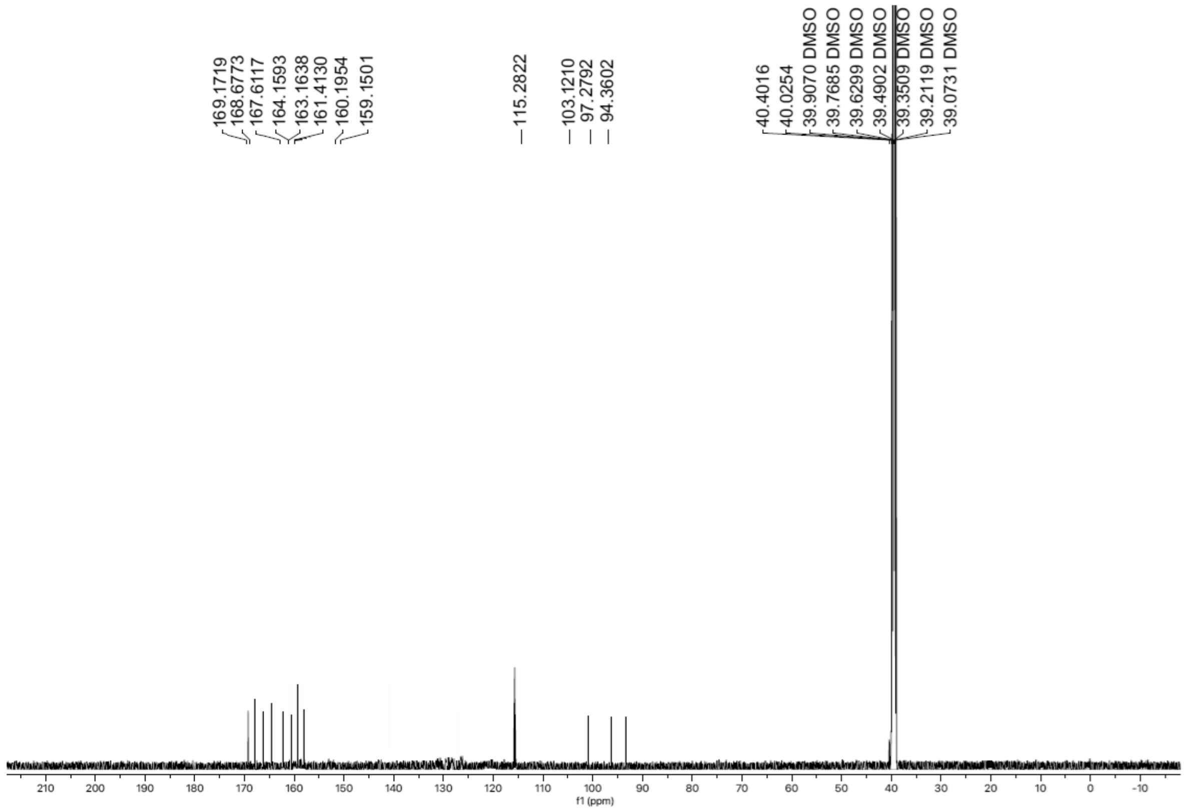

Figure-S.15 Carbon spectral analysis of compound-**3**

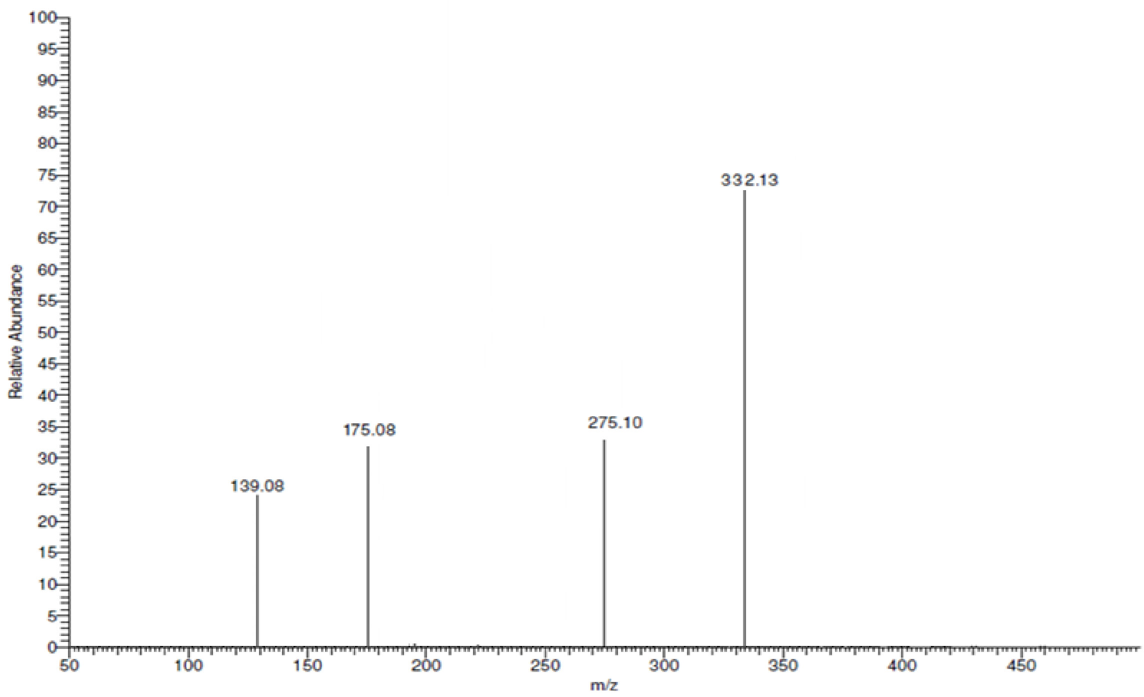

Figure-S.16 HREI-MS spectral analysis of compound-**3**

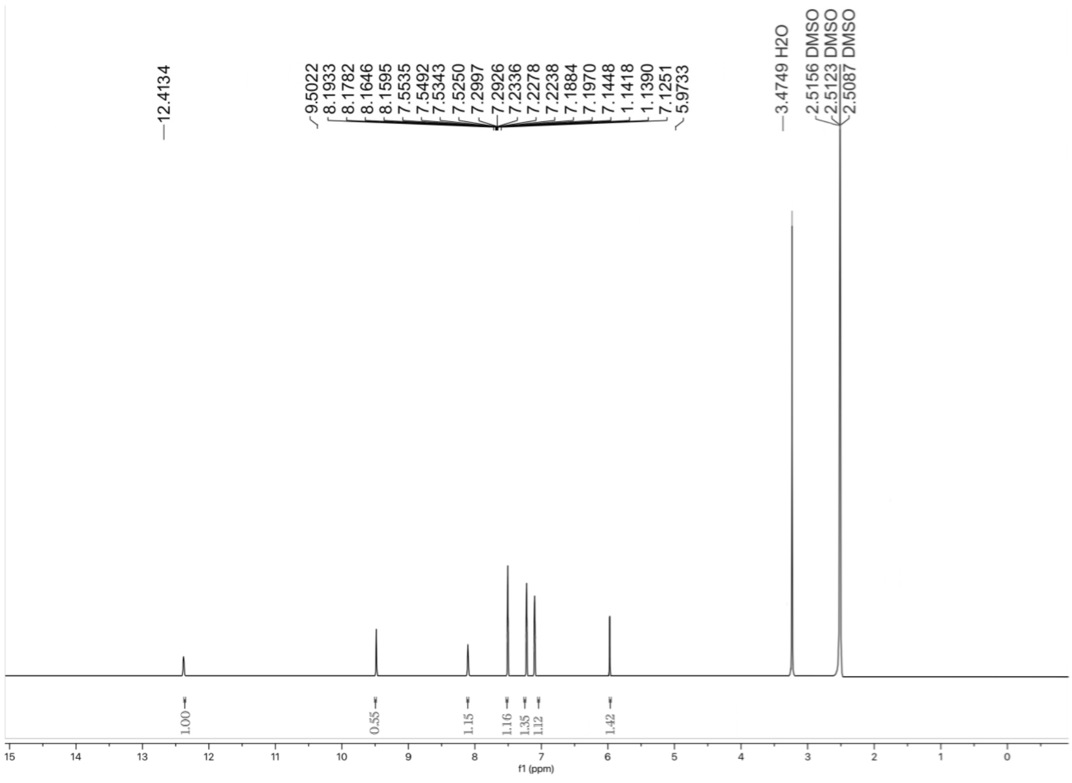

Figure-S.17 Proton spectral analysis of compound-**4**

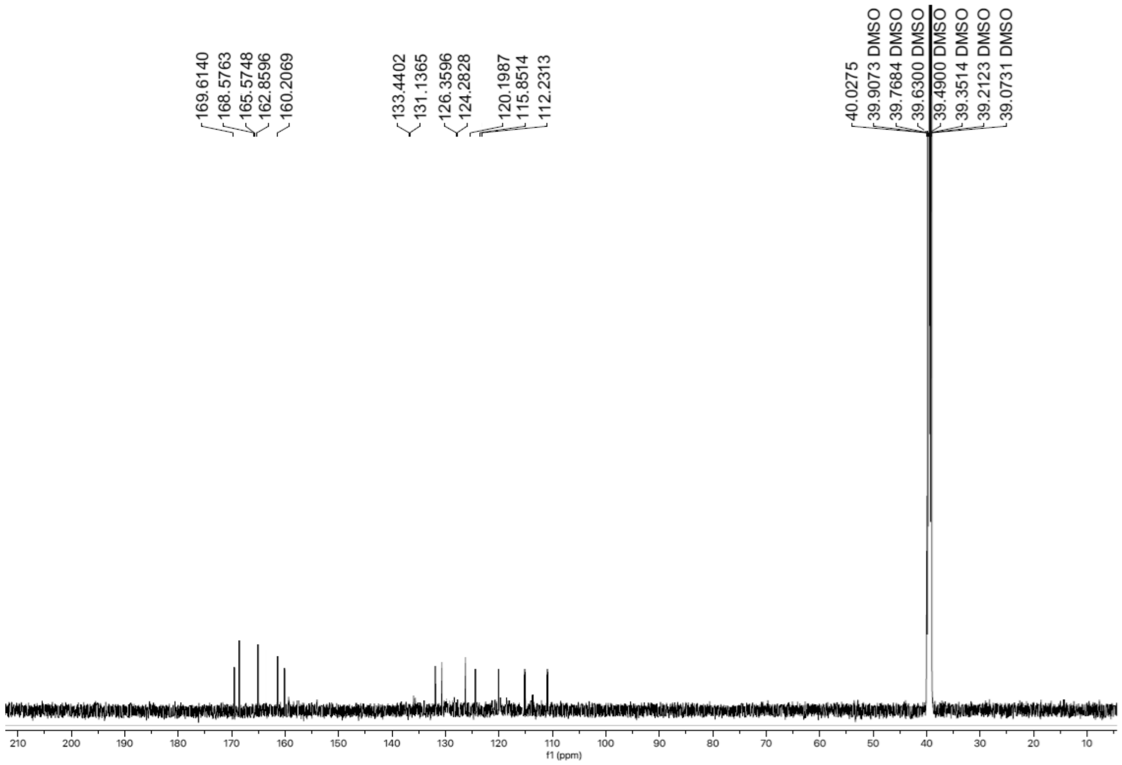

Figure-S.18 Carbon spectral analysis of compound-**4**

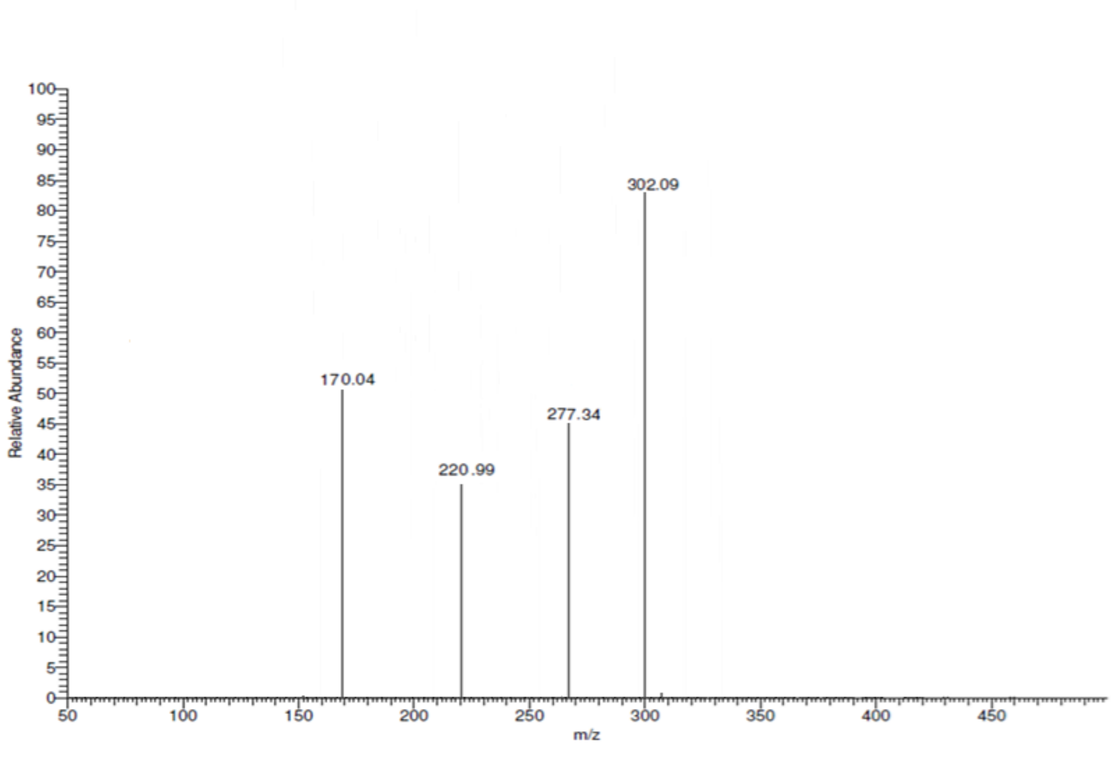

Figure-S.19 HREI-MS spectral analysis of compound-**4**

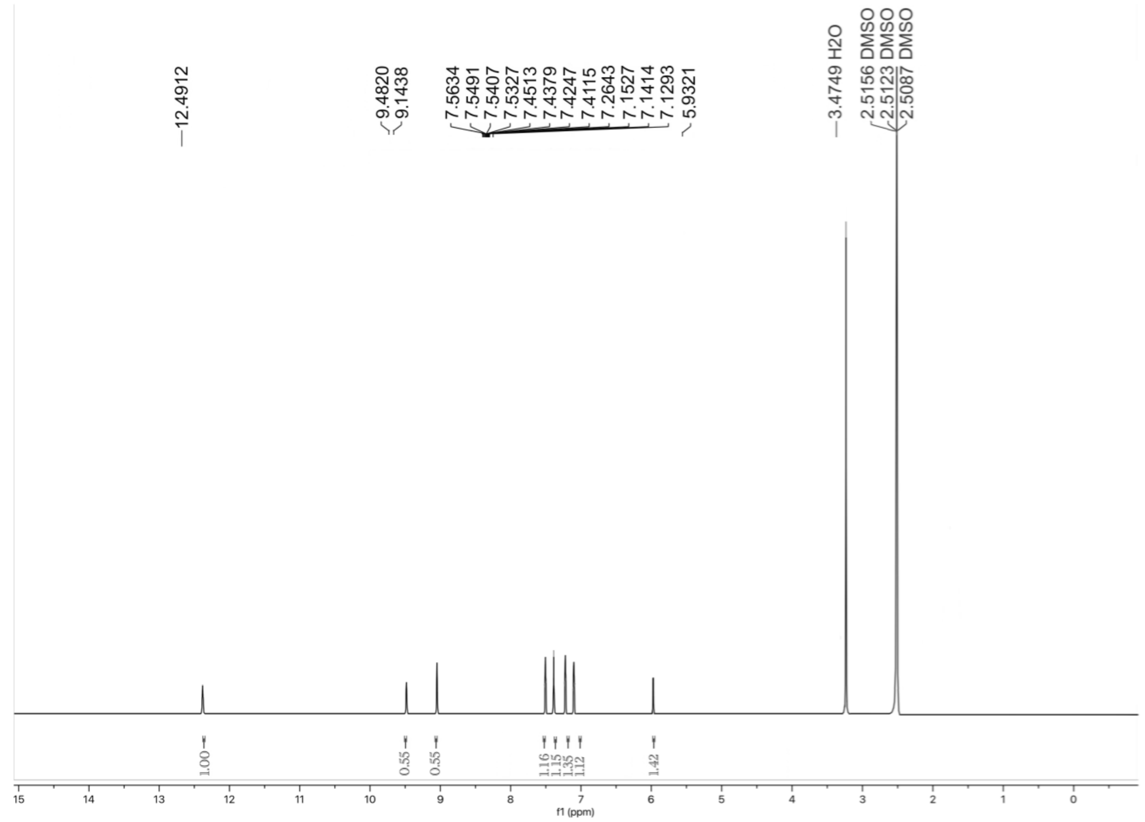

Figure-S.20 Proton spectral analysis of compound-**5**

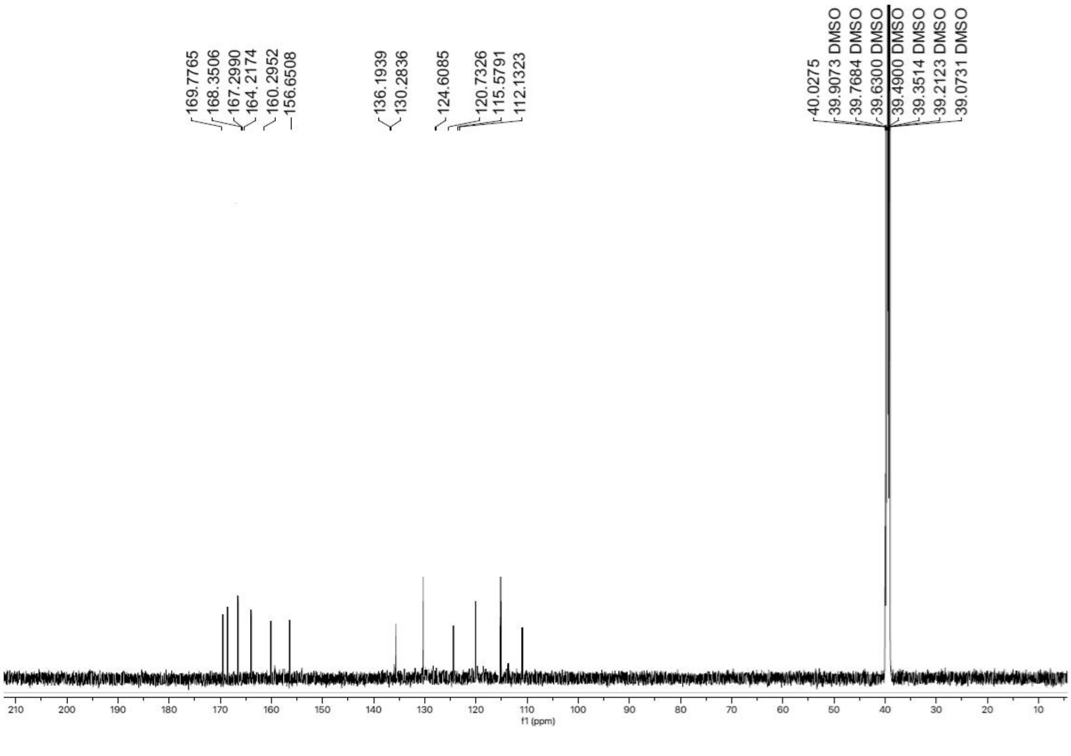

Figure-S.21 Carbon spectral analysis of compound-**5**

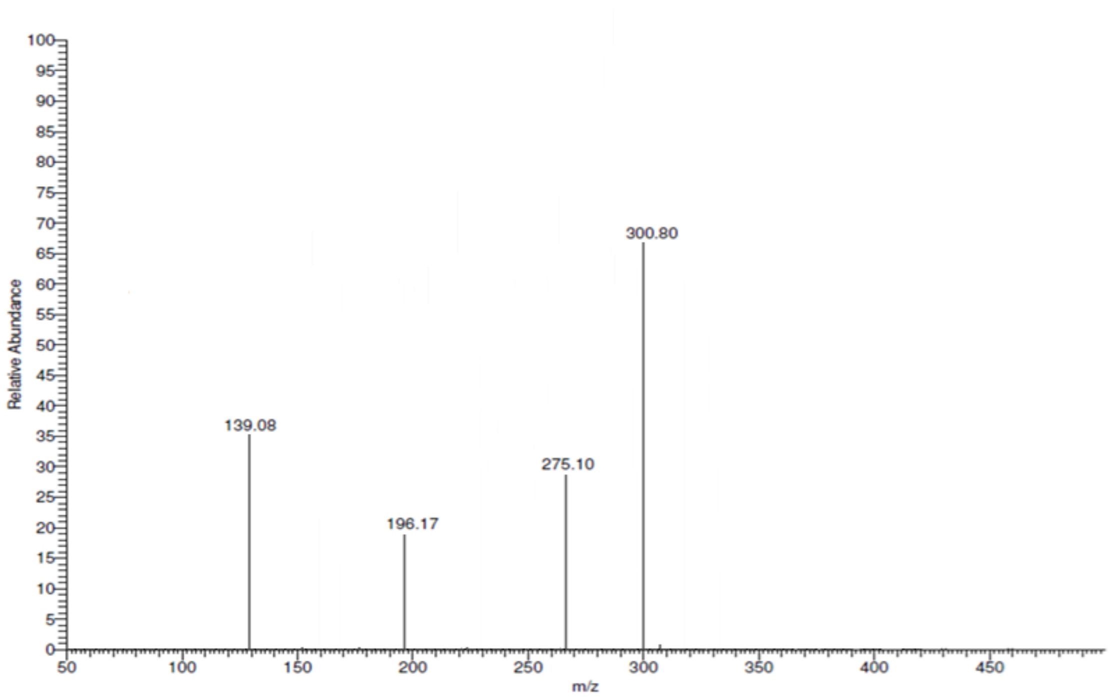

Figure-S.22 HREI-MS spectral analysis of compound-**5**

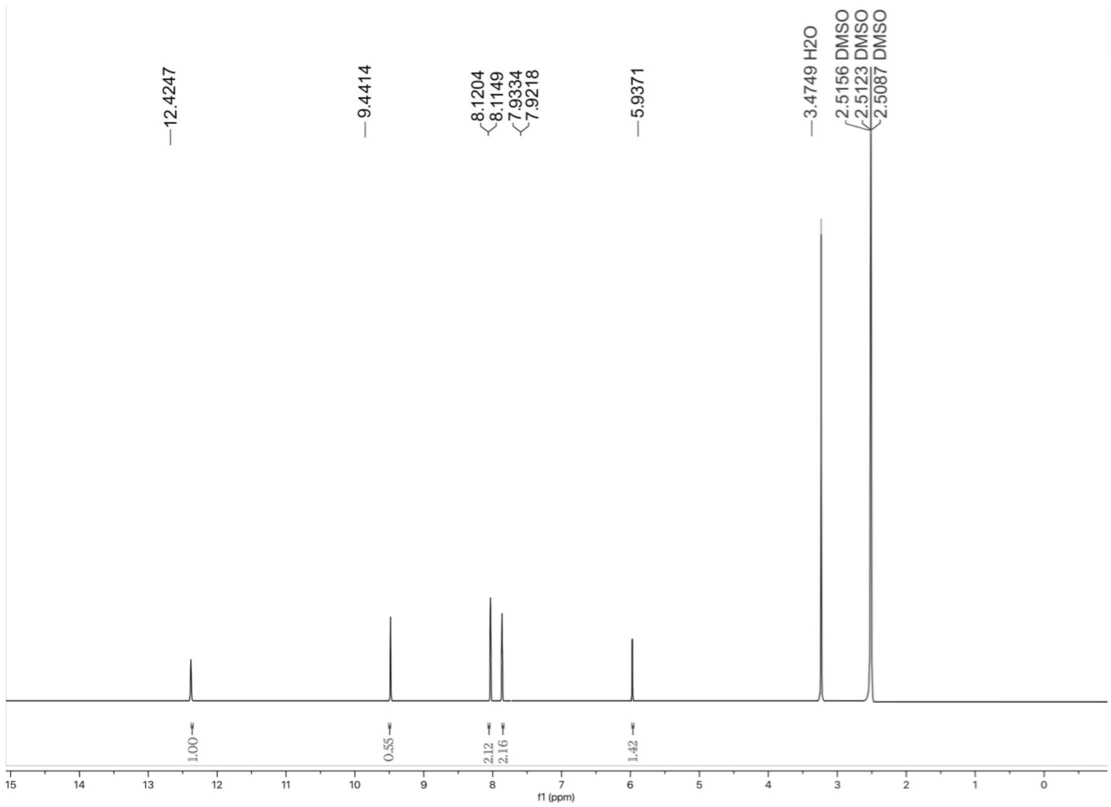

Figure-S.23 Proton spectral analysis of compound-**6**

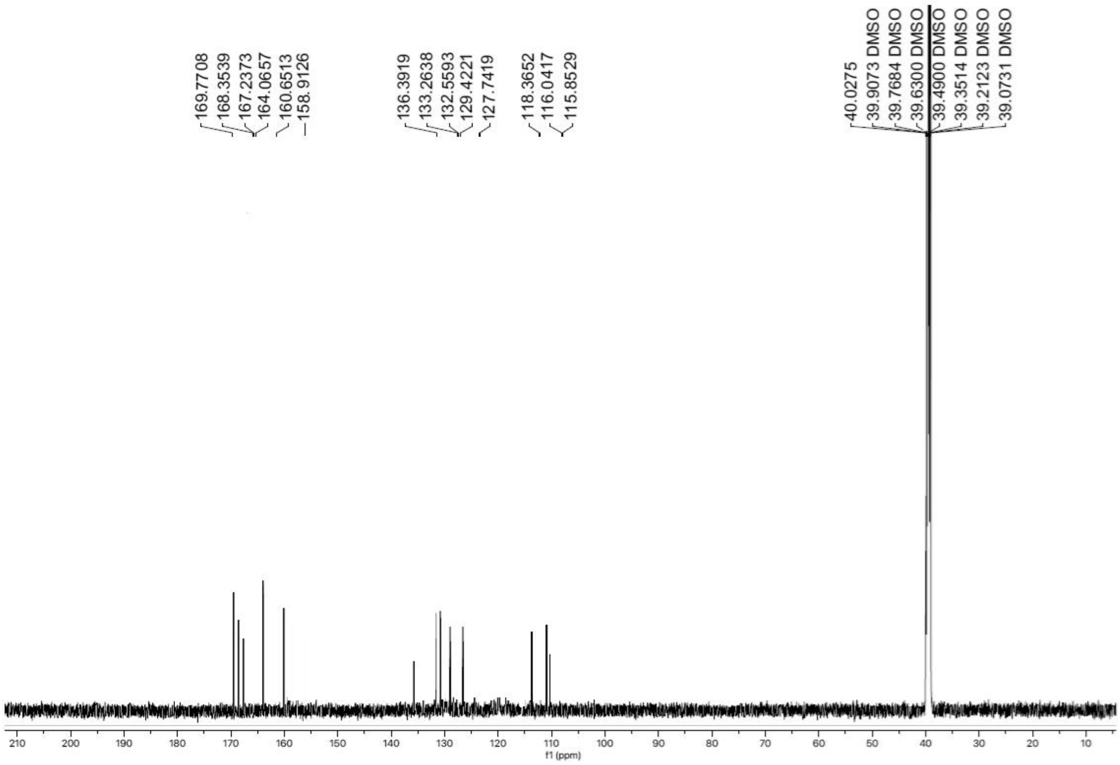

Figure-S.24 Carbon spectral analysis of compound-**6**

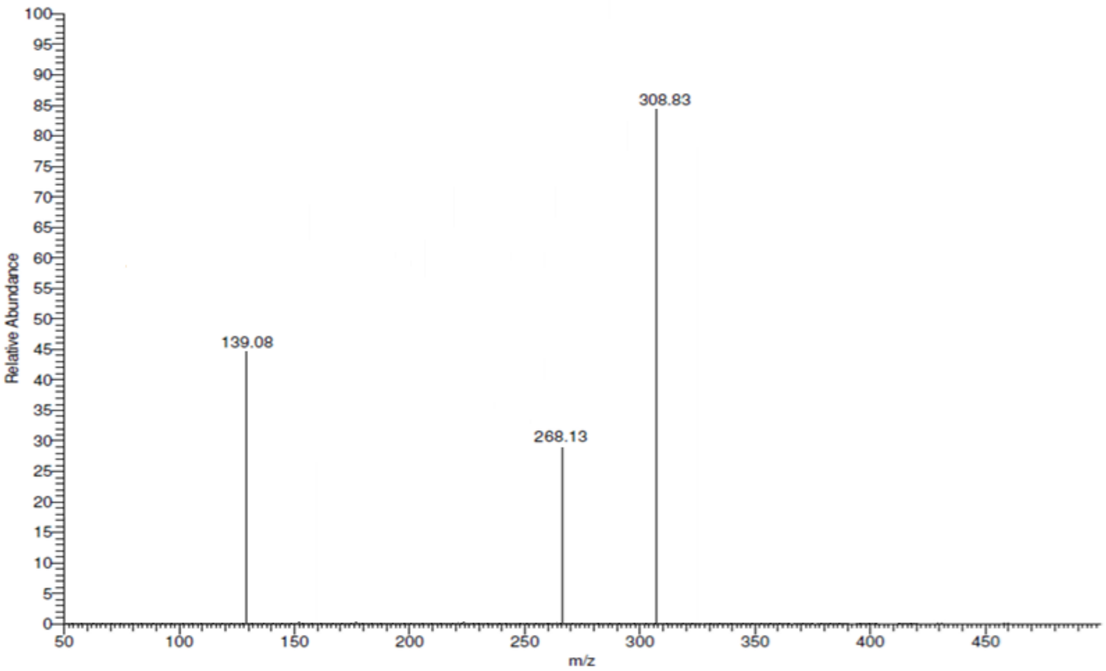

Figure-S.25 HREI-MS spectral analysis of compound-**6**

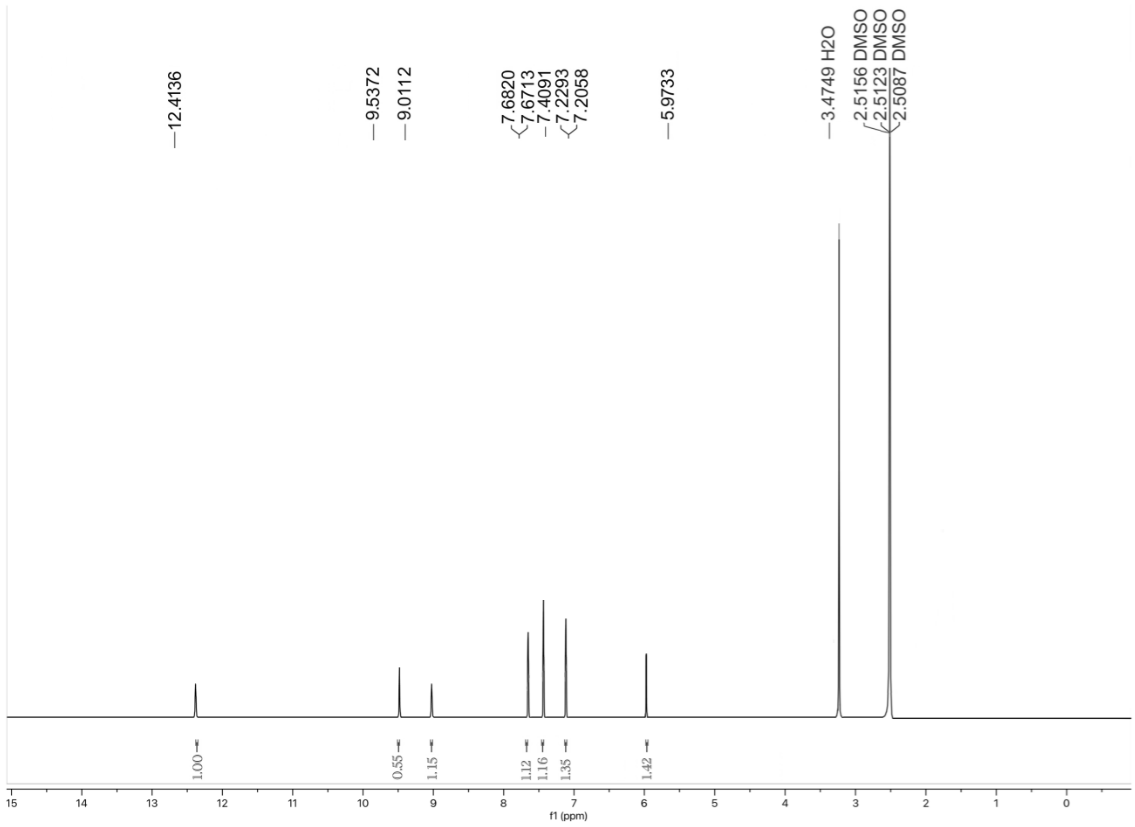

Figure-S.26 Proton spectral analysis of compound-**7**

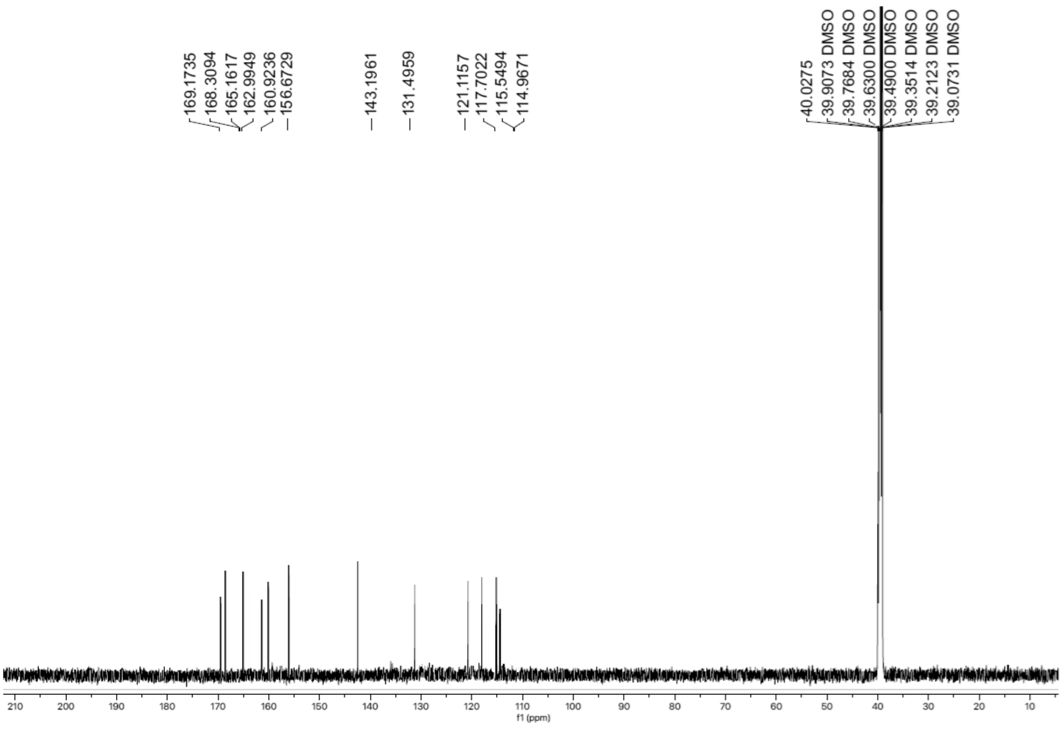

Figure-S.27 Carbon spectral analysis of compound-**7**

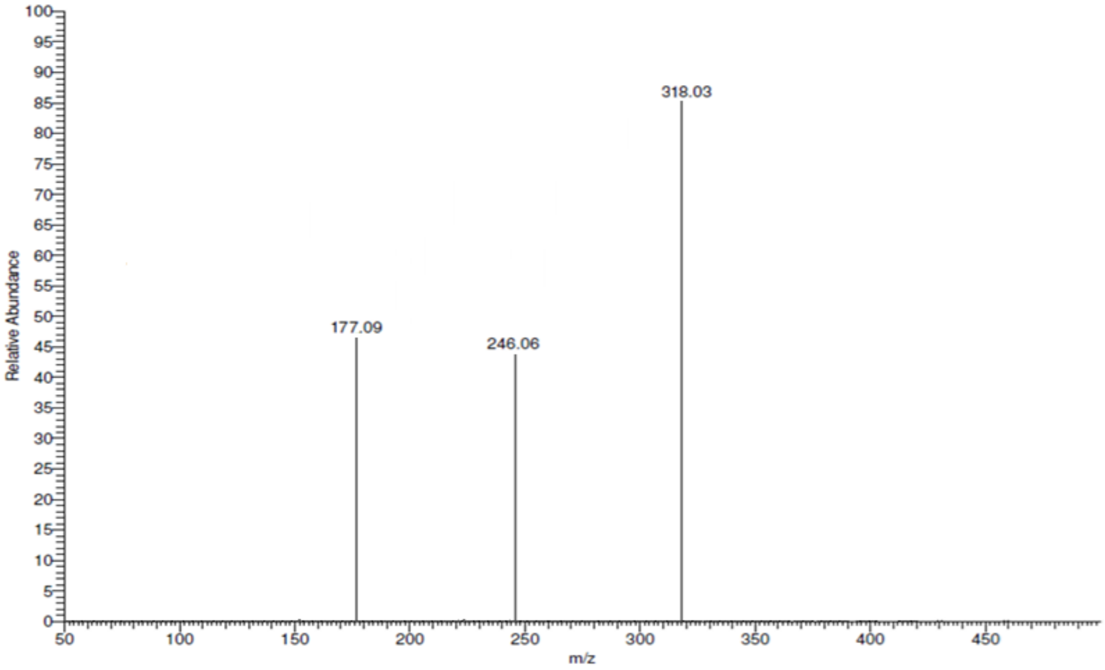

Figure-S.28 HREI-MS spectral analysis of compound-**7**

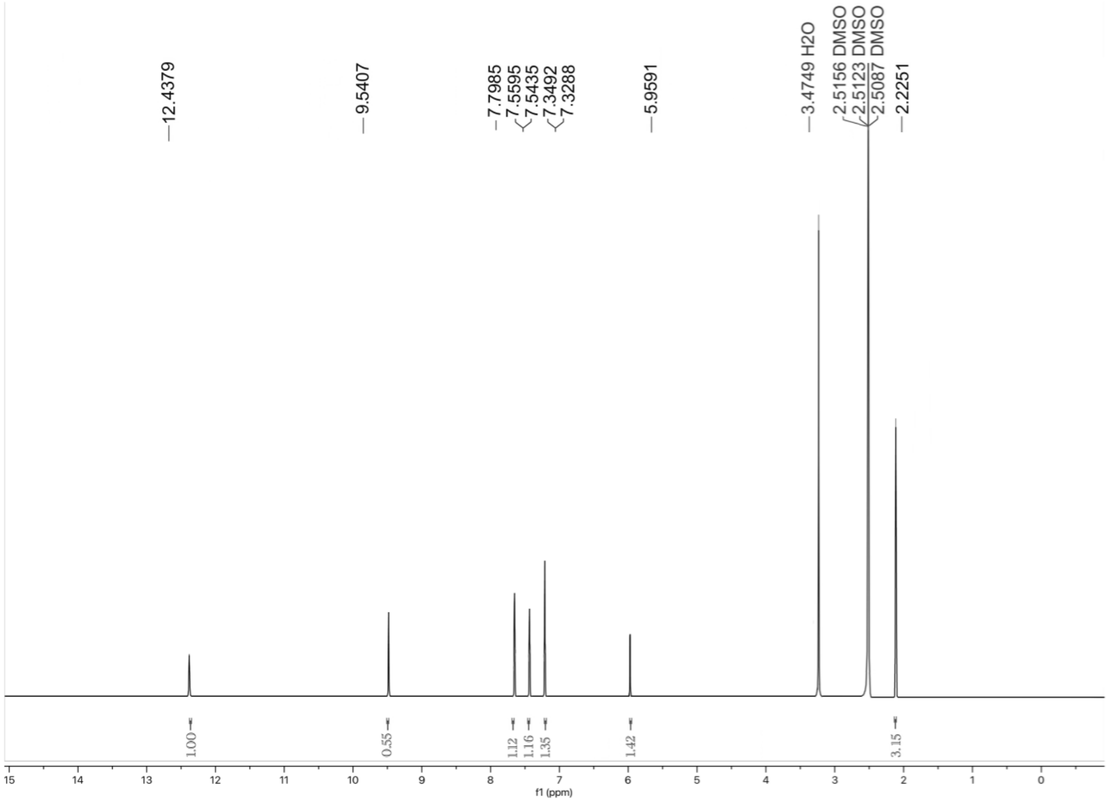

Figure-S.29 Proton spectral analysis of compound-**8**

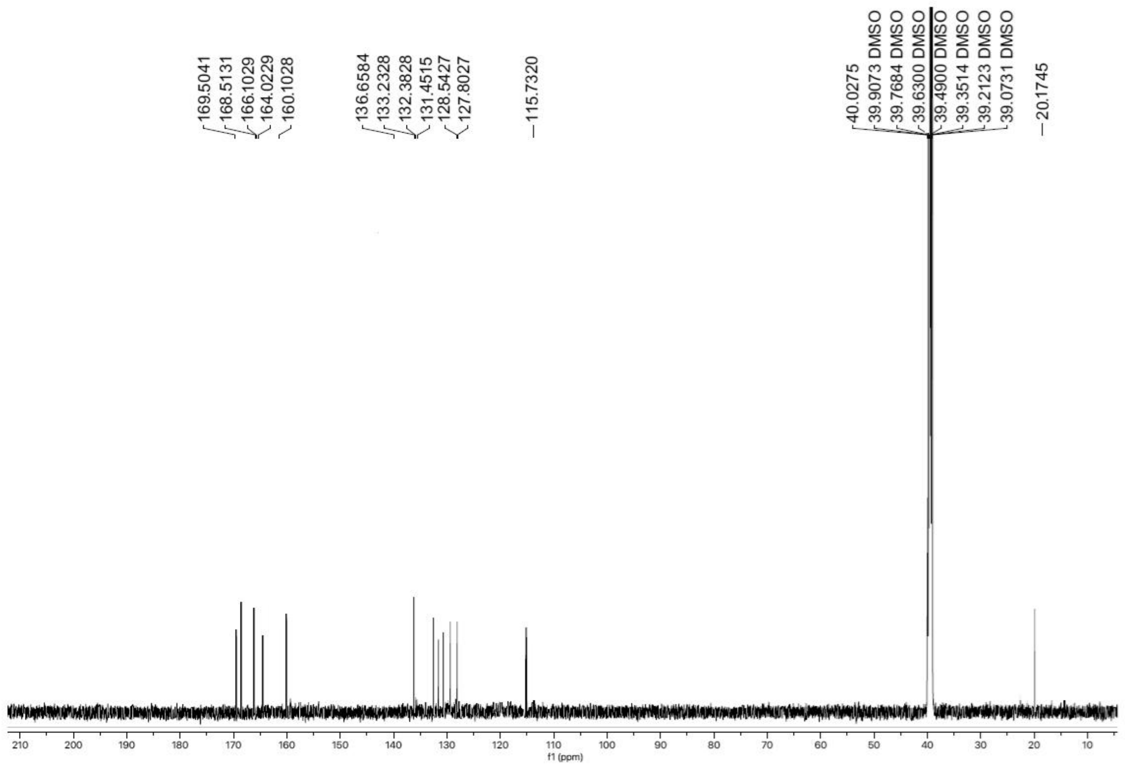

Figure-S.30 Carbon spectral analysis of compound-**8**

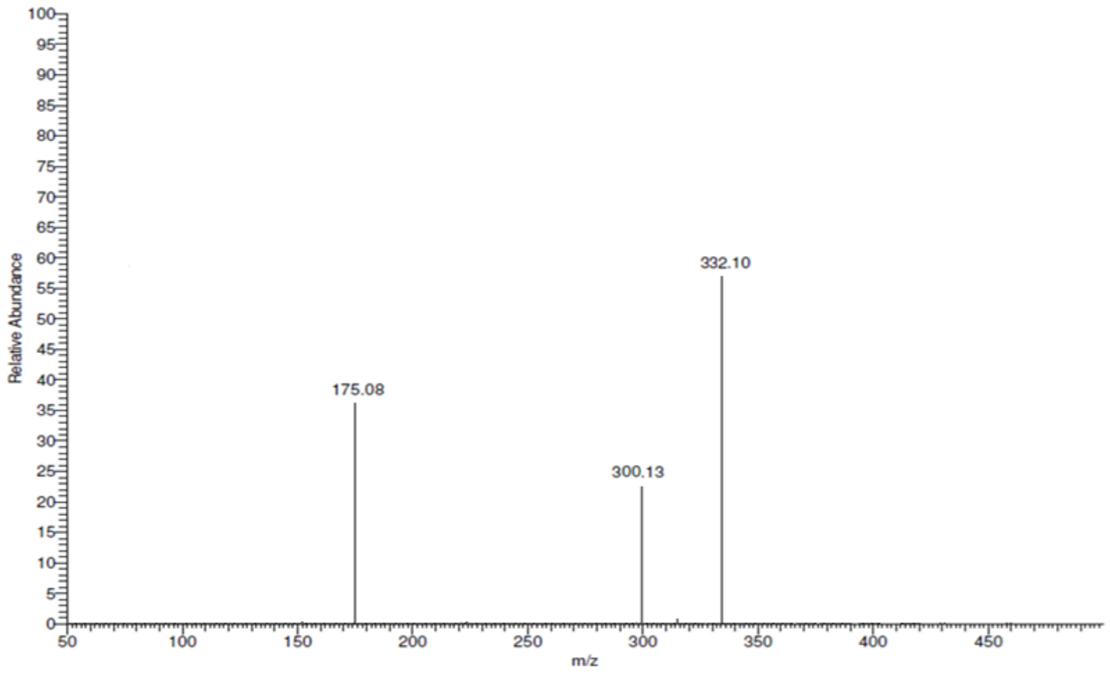

Figure-S.31 HREI-MS spectral analysis of compound-**8**

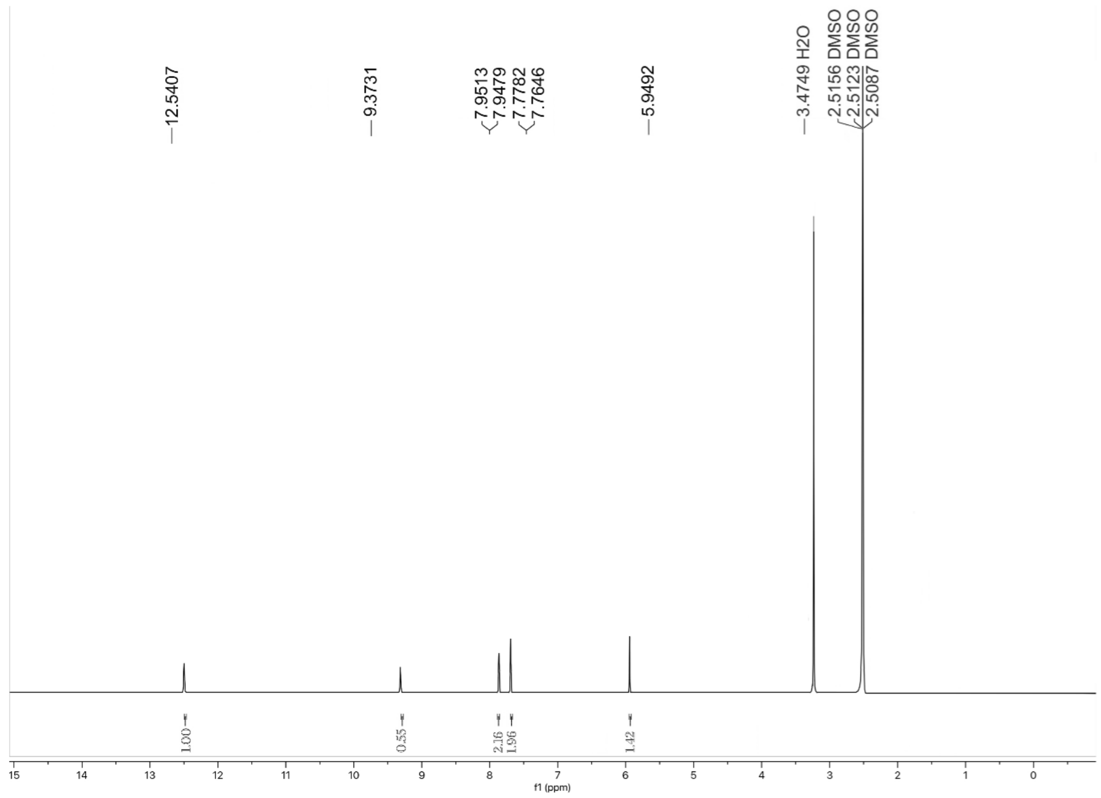

Figure-S.32 Proton spectral analysis of compound-**9**

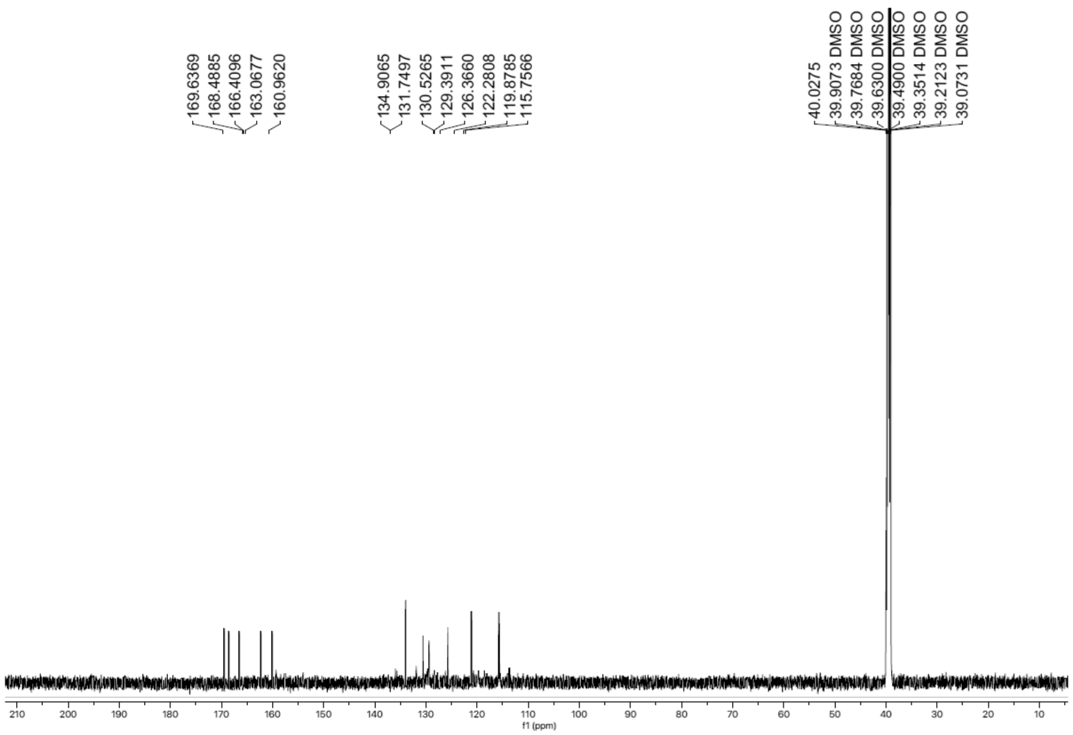

Figure-S.33 Carbon spectral analysis of compound-**9**

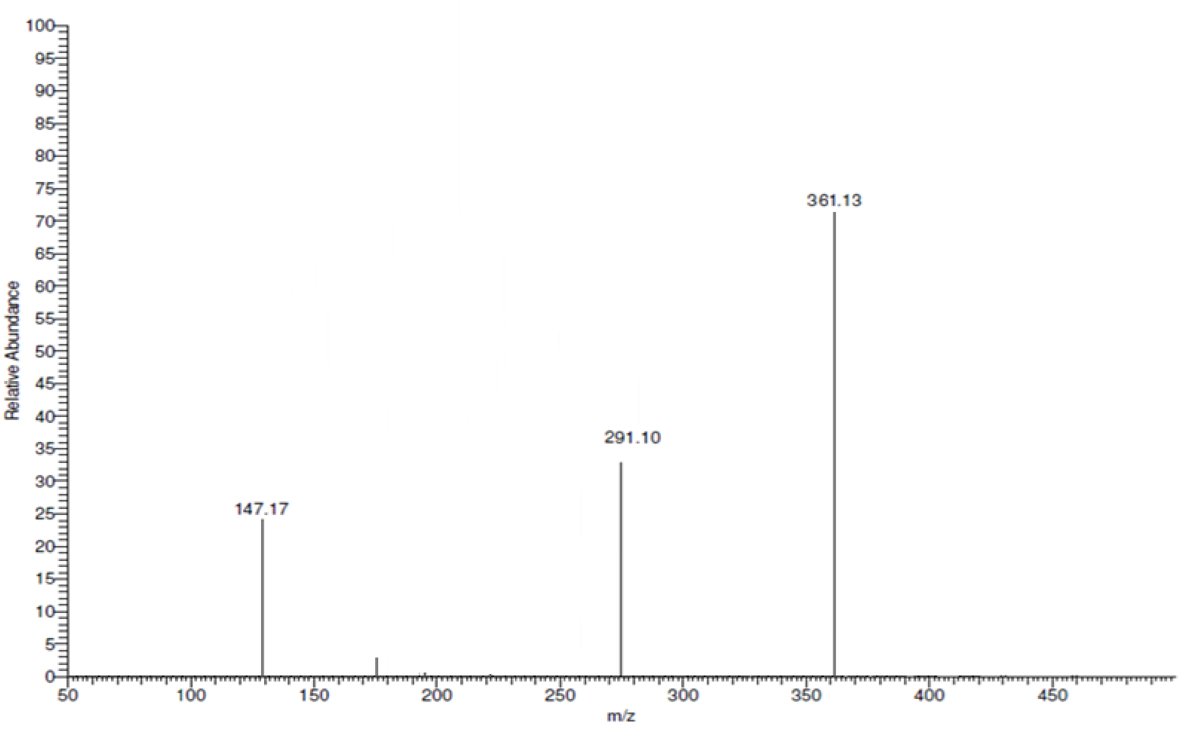

Figure-S.34 HREI-MS spectral analysis of compound-**9**

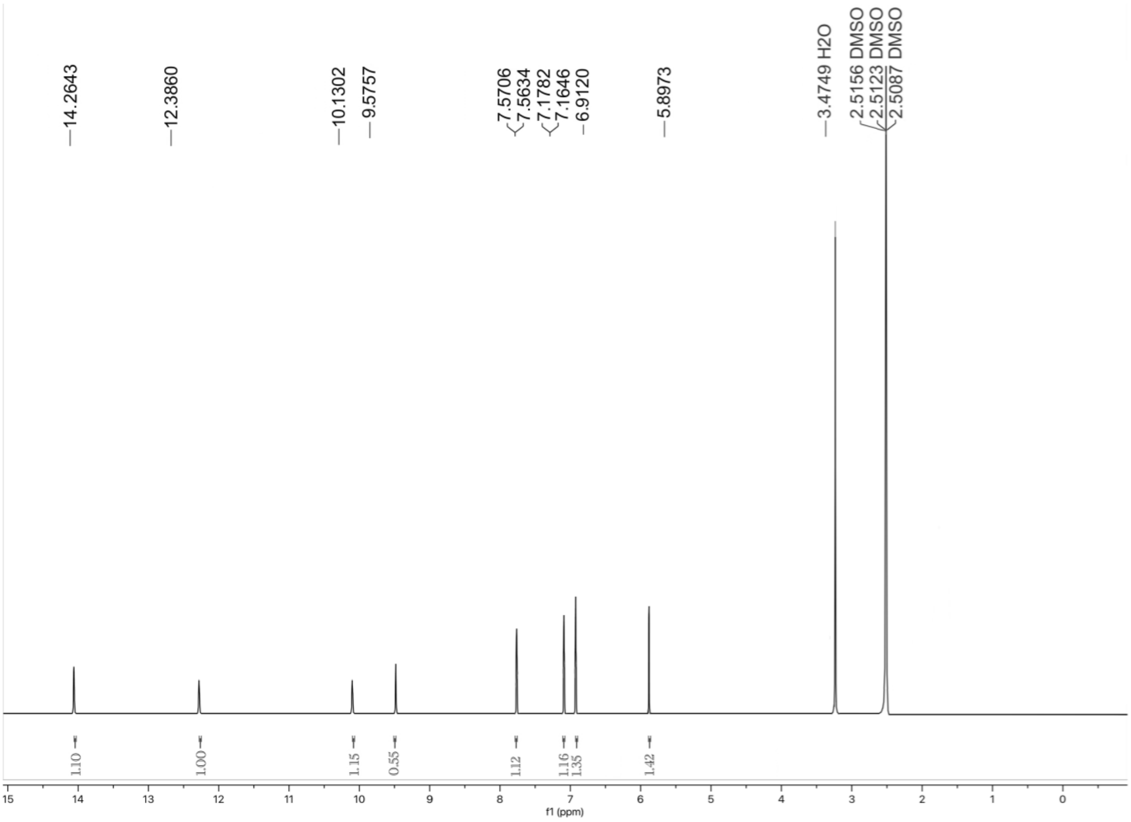

Figure-S.35 Proton spectral analysis of compound-**10**

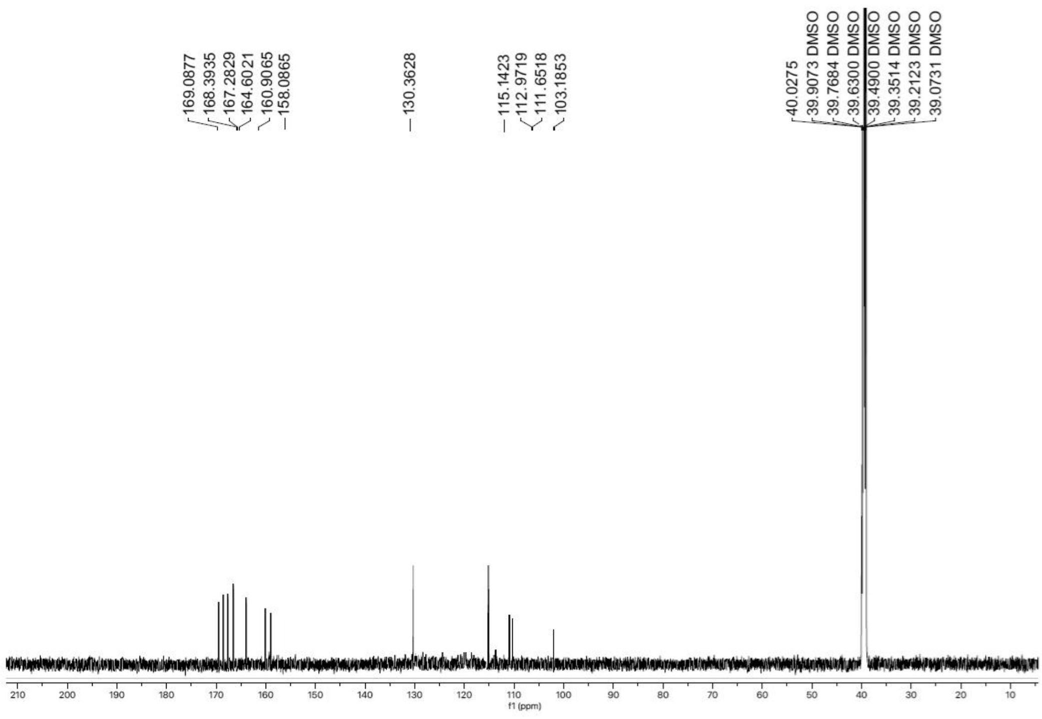

Figure-S.36 Carbon spectral analysis of compound-**10**

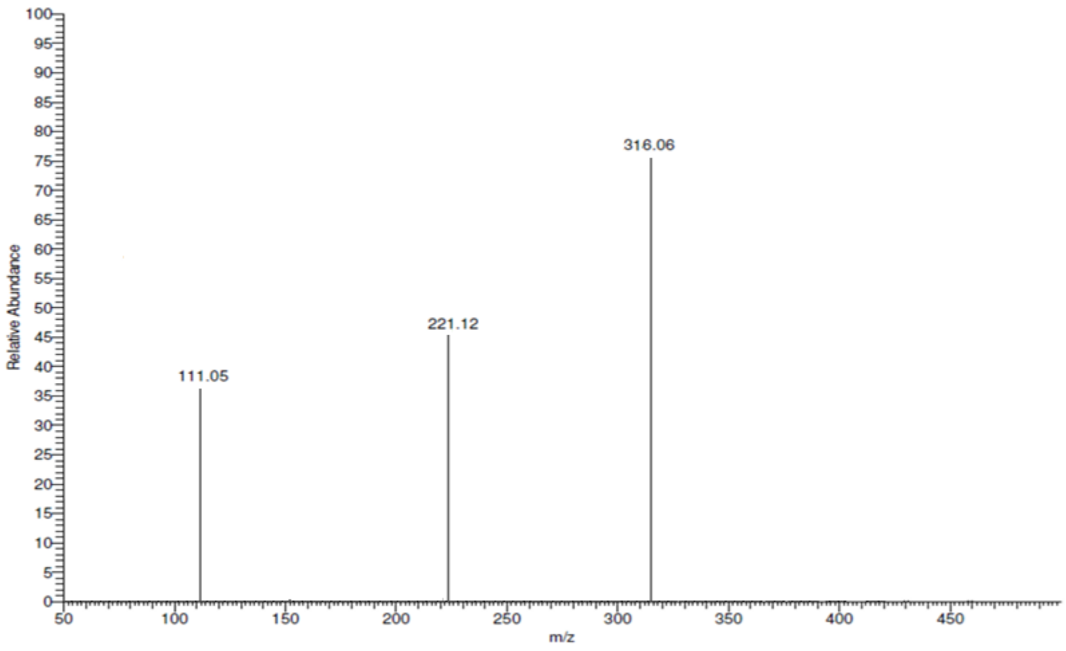

Figure-S.37 HREI-MS spectral analysis of compound-**10**

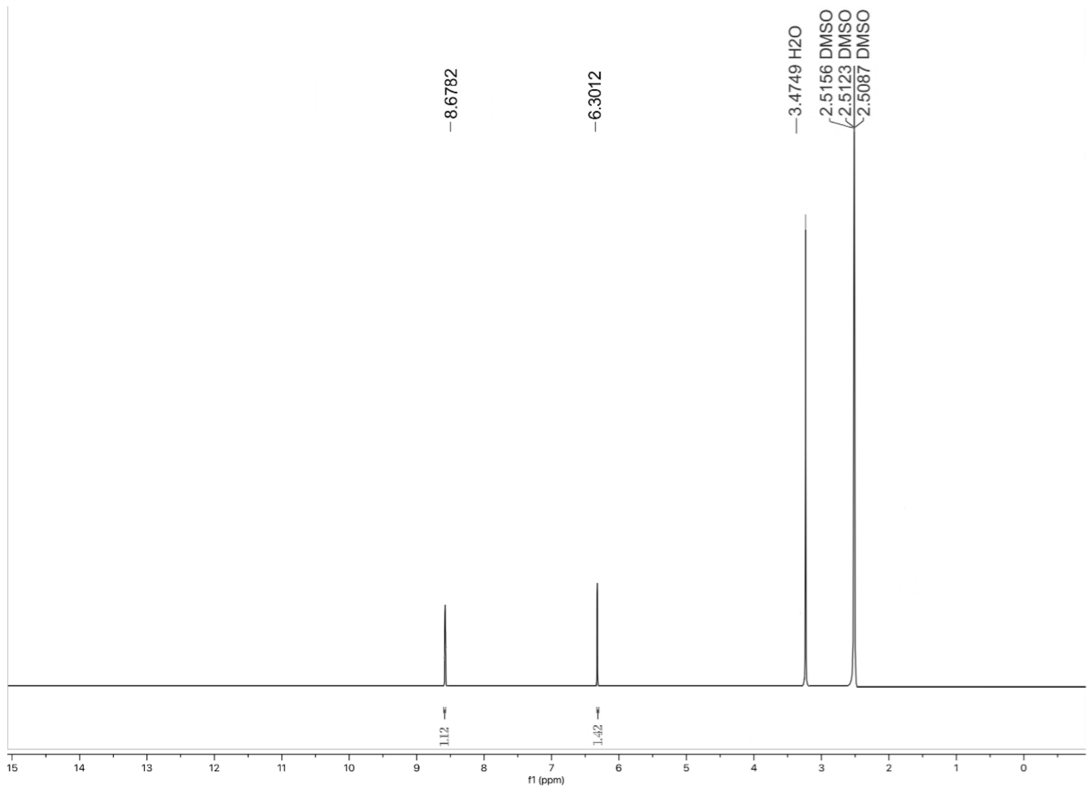

Figure-S.38 Proton spectral analysis of intermediate-1

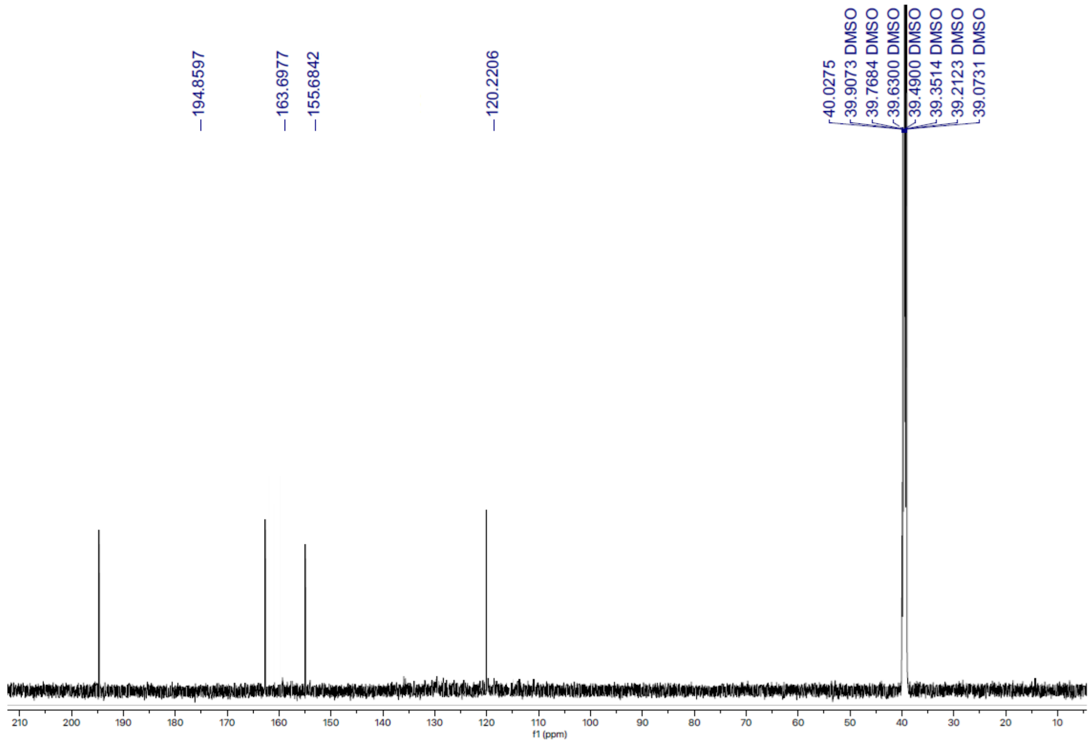

Figure-S.39 Carbon spectral analysis of intermediate-1

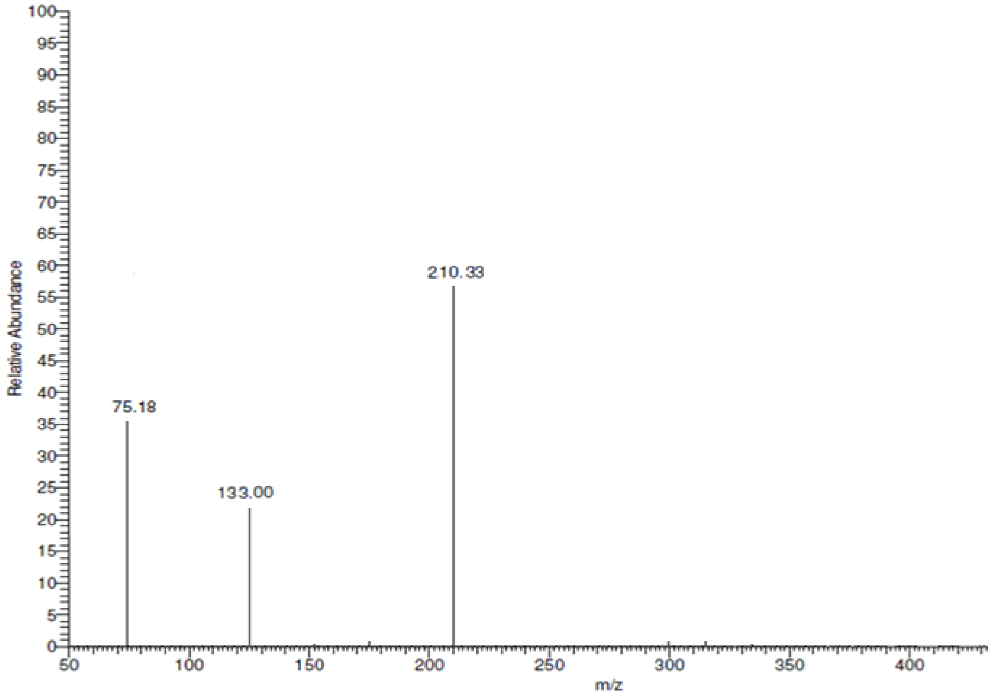

Figure-S.40 HREI-MS spectral analysis of intermediate-1

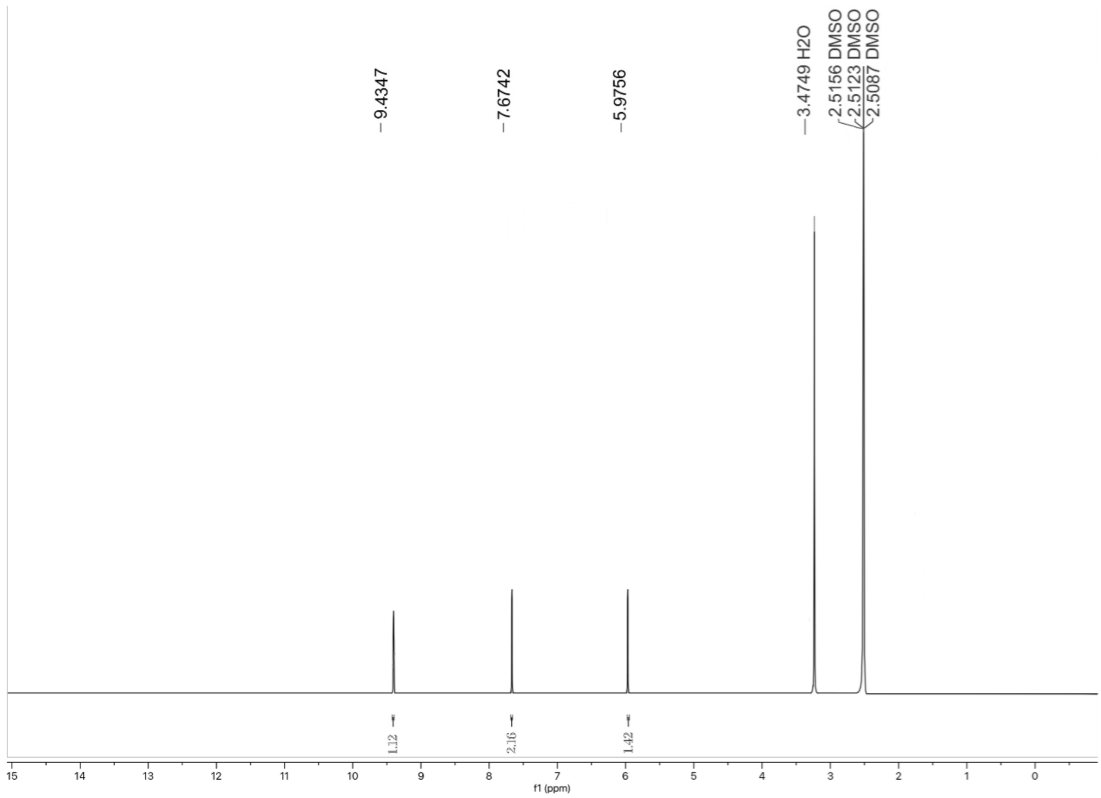

Figure-S.41 Proton spectral analysis of intermediate-2

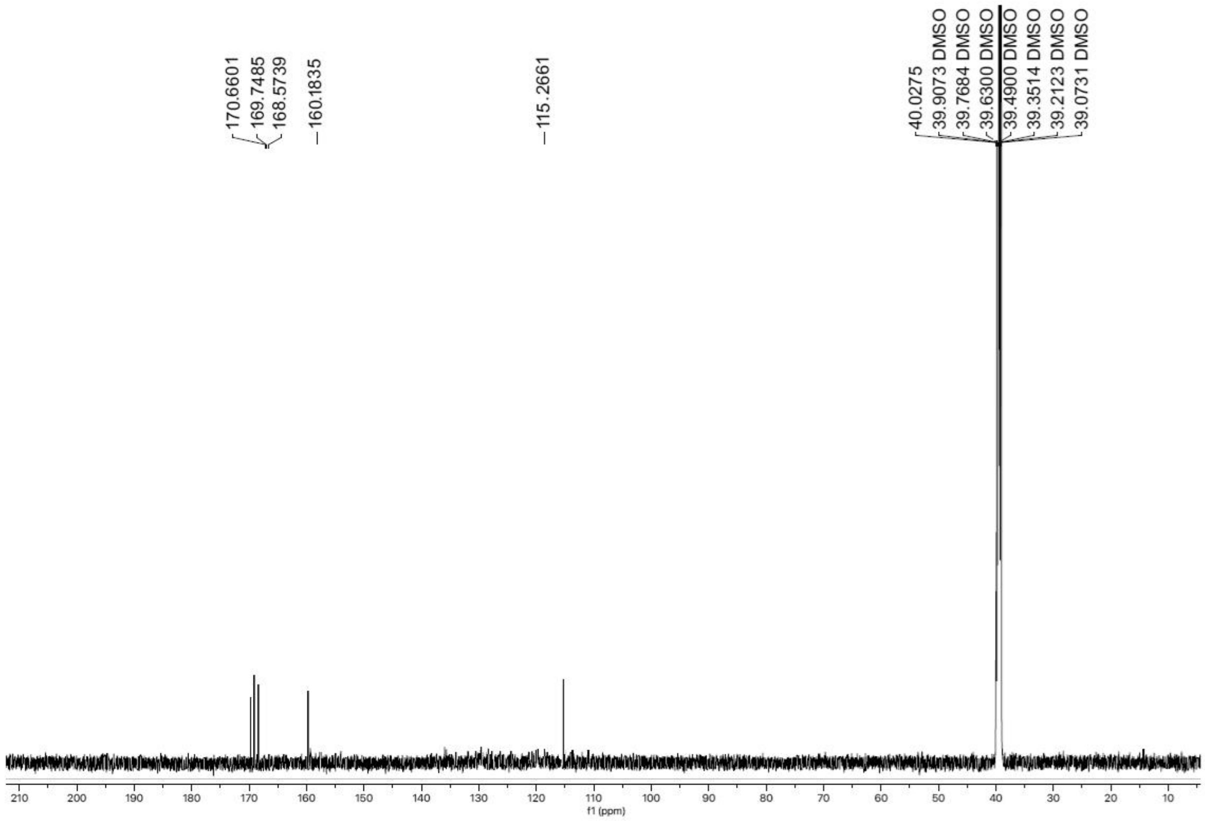

Figure-S.42 Carbon spectral analysis of intermediate-2

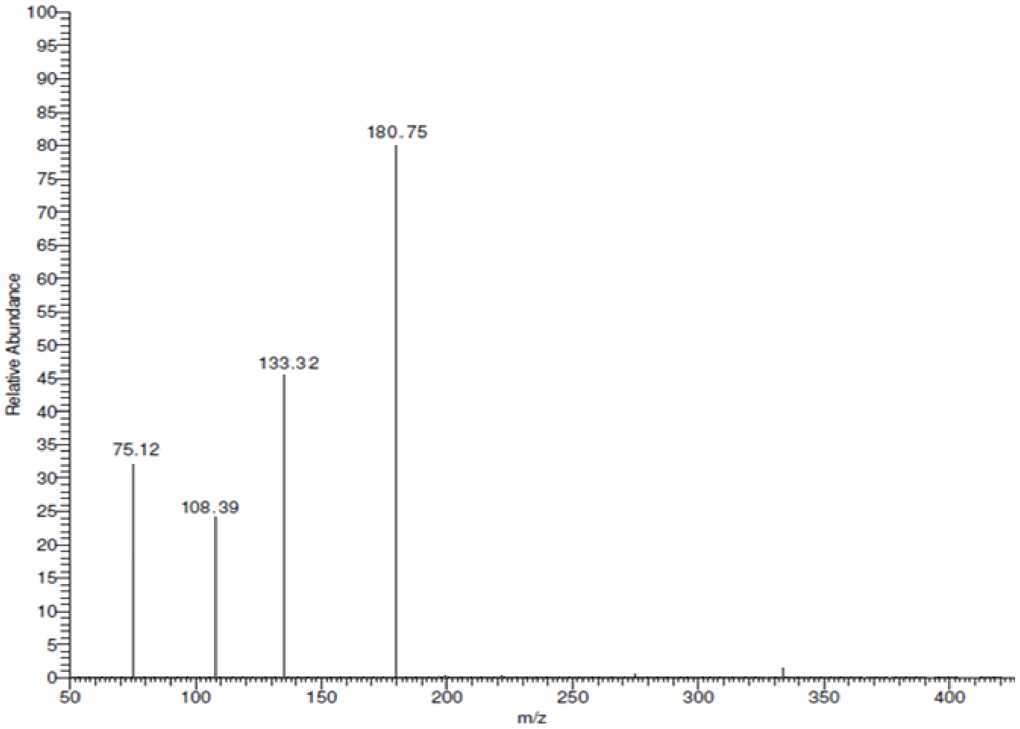

Figure-S.43 HREI-MS spectral analysis of intermediate-2

References

33. Ellman, G.L.; Courtney, K.D.; Andres, J.V.; Featherstone, R.M. A new and rapid colorimetric determination of acetylcholinesterase activity. *Biochem. Pharma.* **1961**, *7*, 88–95.
34. Govindappa, M.; Hemashekhar, B.; Arthikala, M.; Rai, V.R.; Ramachandra, Y.L. Characterization, antibacterial, antioxidant, antidiabetic, antiinflammatory and antityrosinase activity of green synthesized silver nanoparticles using Calophyllum tomentosum leaves extract. *Results Phys.* **2018**, *9*, 400–408.
35. Ali, H.; Houghton, P.J.; Soumyanath, A. α-Amylase inhibitory activity of some Malaysian plants used to treat diabetes; with particular reference to Phyllanthus amarus. *J. Ethnopharmacol.* **2006**, *107*, 449–455.
36. Shoaib, K.; Hussain, R.; Khan, Y.; Iqbal, T.; Ullah, F.; Felemban, S.; Khowdiary. M. M. Facile benzothiazole-triazole based thiazole derivatives as novel thymidine phosphorylase and α-glucosidase inhibitors: Experimental and computational approaches. *Enzyme Microb. Technol.* **2024**, *179*, 110470.
37. Frisch, M.J. Gaussian 98, Revision A.9; Gaussian INc.: Pittsburgh, PA, USA, 1998.
38. El-Baz, A.F.; Sorour, N.M.; Shetaia, Y.M. Trichosporon jirovecii-mediated synthesis of cadmium sulfide nanoparticles. *J. Basic Microbiol.* **2016**, *56*, 520–530.
39. Becke, A.D. Density-functional exchange-energy approximation with correct asymptotic behavior. *Phys. Rev. A* **1988**, *38*, 3098–3100.
40. Lee, C.; Yang, W.; Parr, R.G. Development of the Colle-Salvetti correlation-energy formula into a functional of the electron density. *Phys. Rev. B* **1988**, *37*, 785–789.
41. Flurry, R.L., Jr. Molecular Orbital Theory of Bonding in Organic Molecules; Marcel Dekker: New York, NY, USA, 1968.
42. Seboletswe, P.; Kumar, G.; Gcabashe, N.; Olofinsan, K.; Idris, A.; Islam, S.; Idris, A.; Singh, P.; Benzylidenehydrazine Derivatives: Synthesis, Antidiabetic Evaluation, Antioxidation, Mode Of Inhibition, DFT And Molecular Docking Studies. *Chem. Biodivers.* **2025**, *22*, e202401556.
43. Rehman, M.T.; AlAjmi, M.F.; Hussain, A.; Rather, G.M.; Khan, M.A. High-Throughput Virtual Screening, Molecular Dynamics Simulation, and Enzyme Kinetics Identified ZINC84525623 as a Potential Inhibitor of NDM-1. *Int. J. Mol. Sci.* **2019**, *20*, 819.
44. AlAjmi, M.F.; Rehman, M.T.; Hussain, A.; Rather, G.M. Pharmacoinformatics approach for the identification of Polo-like kinase-1 inhibitors from natural sources as anti-cancer agents. *Int. J. Biol. Macromol.* **2018**, *116*, 173–181.
45. Brańka, A.C. Nosé-Hoover chain method for nonequilibrium molecular dynamics simulation. *Phys. Rev. E* **2000**, *61*, 4769–4773.
46. Martyna, G.J.; Tobias, D.J.; Klein, M.L. Constant pressure molecular dynamics algorithms. *J. Chem. Phys.* **1994**, *101*, 4177–4189.
